# Mechanisms through which lithocholic acid delays yeast chronological aging under caloric restriction conditions

**DOI:** 10.18632/oncotarget.26188

**Published:** 2018-10-09

**Authors:** Anthony Arlia-Ciommo, Anna Leonov, Karamat Mohammad, Adam Beach, Vincent R. Richard, Simon D. Bourque, Michelle T. Burstein, Alexander A. Goldberg, Pavlo Kyryakov, Alejandra Gomez-Perez, Olivia Koupaki, Vladimir I. Titorenko

**Affiliations:** ^1^ Department of Biology, Concordia University, Montreal, Quebec, Canada

**Keywords:** yeast, cellular aging, geroprotectors, lithocholic acid, metabolism

## Abstract

All presently known geroprotective chemical compounds of plant and microbial origin are caloric restriction mimetics because they can mimic the beneficial lifespan- and healthspan-extending effects of caloric restriction diets without the need to limit calorie supply. We have discovered a geroprotective chemical compound of mammalian origin, a bile acid called lithocholic acid, which can delay chronological aging of the budding yeast *Saccharomyces cerevisiae* under caloric restriction conditions. Here, we investigated mechanisms through which lithocholic acid can delay chronological aging of yeast limited in calorie supply. We provide evidence that lithocholic acid causes a stepwise development and maintenance of an aging-delaying cellular pattern throughout the entire chronological lifespan of yeast cultured under caloric restriction conditions. We show that lithocholic acid stimulates the aging-delaying cellular pattern and preserves such pattern because it specifically modulates the spatiotemporal dynamics of a complex cellular network. We demonstrate that this cellular network integrates certain pathways of lipid and carbohydrate metabolism, some intercompartmental communications, mitochondrial morphology and functionality, and liponecrotic and apoptotic modes of aging-associated cell death. Our findings indicate that lithocholic acid prolongs longevity of chronologically aging yeast because it decreases the risk of aging-associated cell death, thus increasing the chance of elderly cells to survive.

## INTRODUCTION

The budding yeast *Saccharomyces cerevisiae* is a unicellular eukaryote that has been successfully used as a model organism to identify genes and signaling pathways involved in aging; after being discovered in *S. cerevisiae*, these genes and signaling pathways have been shown to affect cellular and organismal aging also in multicellular eukaryotes across phyla [1–5]. *S. cerevisiae* is a valuable model organism for unveiling mechanisms of aging and longevity because both replicative and chronological lifespan assays in this yeast are amenable to thorough genetic, biochemical, cell biological, chemical biological and system biological analyses [3–11]. Studies in *S. cerevisiae* showed that the key aspects of the aging process have been conserved during evolution [1–5, 8–29]. These evolutionarily conserved aspects include mechanisms through which some dietary regimens and certain chemical compounds can slow down the aging process [1–5, 10, 18–29].

One of the aging-delaying dietary regimens is caloric restriction (CR), which limits calorie supply without restricting the supply of amino acids and other nutrients [1, 18, 19]. CR has been shown to slow down the replicative and chronological modes of aging in yeast [1, 3, 5, 15], and to extend healthspan by decelerating the aging process in evolutionarily distant eukaryotic organisms [1, 18, 19]. In multicellular eukaryotes across phyla, organismal aging can be delayed, and the onset of aging-associated diseases can be postponed not only by CR but also by certain chemical compounds of plant and microbial origin. These geroprotective chemical compounds include resveratrol, rapamycin, curcumin, fisetin, quercetin, caffeine and spermidine; all of them exhibit beneficial effects on organismal lifespan and healthspan only under non-CR conditions [1, 19–29]. All these aging-delaying chemical compounds of plant and microbial origin have been discovered by studies in yeast. In *S. cerevisiae*, these geroprotectors slow down the replicative and chronological modes of aging under non-CR conditions by controlling information flow along a network that integrates an evolutionarily conserved set of signaling pathways and protein kinases [1, 19–26, 30–43].

We have discovered a geroprotective chemical compound of mammalian origin, a bile acid called lithocholic acid (LCA) [36]. It delays yeast chronological aging mainly under CR conditions and only if exogenously added at certain critical periods of lifespan [36, 44]. Our previous studies have demonstrated that exogenous LCA enters yeast cells limited in calorie supply, is sorted to mitochondria, amasses primarily in the inner mitochondrial membrane (IMM) and also resides in the outer mitochondrial membrane (OMM), alters the concentrations of certain mitochondrial membrane phospholipids, elicits a major enlargement of mitochondria, significantly decreases mitochondrial number, prompts an intra-mitochondrial accumulation of cristae disconnected from the IMM, triggers substantial alterations in mitochondrial proteome, decreases the frequencies of deletion and point mutations in mitochondrial DNA (mtDNA), and leads to changes in vital aspects of mitochondrial functionality [36, 44–54]. We have provided evidence that the LCA-dependent regulation of these mitochondrial processes makes an essential contribution to the LCA-dependent delay of yeast chronological aging under CR conditions [36, 44–54].

As detailed below, our recent studies have indicated that LCA influences not only the aforesaid processes in mitochondria but also some other cellular processes that take place in several cellular compartments. Based on these recent studies, we hypothesized that LCA may delay chronological aging of yeast limited in calorie supply also because it affects these other cellular processes confined to various cellular locations. This study is aimed at testing our hypothesis by investigating mechanisms through which LCA controls the spatiotemporal dynamics of these other cellular processes in different cellular locations under CR conditions. We also examined if there is a causal relationship between such LCA-driven control of these other cellular processes and the LCA-dependent deceleration of yeast chronological aging when calorie supply is limited. In support of our hypothesis, we show that LCA stimulates an aging-delaying cellular pattern and preserves such pattern throughout the entire chronological lifespan of yeast because under CR conditions it alters the spatiotemporal dynamics of these other cellular processes confined to several cellular locations.

LCA is known to play essential roles in the maintenance of a heathy lifespan in evolutionarily distant organisms. Specifically, LCA has been shown to extend the organismal lifespan in fruit flies [55]. LCA is also known to exhibit potent and specific anti-tumor effects in cultured human cancer cells [56–61], with cancer considered to be an aging-associated disease [47, 62–71]. Because this study provides important new insights into the mechanisms through which LCA delays the chronological aging process in yeast, it advances our knowledge of the mechanisms underlying the lifespan-extending and healthspan-improving effects of LCA in humans and other multicellular eukaryotic organisms.

## RESULTS

### Out hypothesis on mechanisms through which LCA may delay yeast chronological aging under CR conditions

Our recent studies have revealed that LCA not only alters mitochondrial lipidome and proteome, enlarges mitochondria, decreases mitochondrial number, modifies mitochondrial cristae structure, and changes vital aspects of mitochondrial functionality [36, 44–54], but also affects some other cellular processes in different cellular locations. Specifically, during post-diauxic (PD) and the non-proliferative stationary (ST) phases of culturing yeast under CR conditions on 0.2% glucose, LCA also affects the following cellular processes: 1) it increases the concentration of triacylglycerols (TAG), so-called ″neutral″ (uncharged) lipids initially synthesized in the endoplasmic reticulum (ER) and then deposited in lipid droplets (LD) (Supplementary Figure 1); 2) it decreases the concentration of free fatty acids (FFA), which can be used as substrates for TAG synthesis in the ER and can also be formed as the products of TAG lipolysis in LD; FFA can then undergo β-oxidation in peroxisomes (Supplementary Figure 1); 3) it increases the concentrations of the Gpd2, Gpt2, Slc1, Are1 and Dga1 proteins, all of which reside in the ER and are involved in the synthesis of TAG from FFA (Supplementary Figure 1); 4) it decreases the concentrations of the Tgl1, Tgl3, Tgl4 and Tgl5 proteins, all of which exist in LD and catalyze TAG lipolysis that yields FFA (Supplementary Figure 1); 5) it increases the concentrations of Cat2, Crc1, Yat1 and Yat2; these proteins are required for the carnitine-dependent transport of acetyl-CoA from peroxisomes (where acetyl-CoA is formed as the final product of the β-oxidation of FFA) to mitochondria (Supplementary Figure 2); 6) it decreases the concentrations of the Cit2, Ctp1 and Dic1 proteins (Cit2 catalyzes a peroxisomal anaplerotic reaction transforming acetyl-CoA into citrate; this reaction is followed by the conversion of citrate into succinate and then by the Ctp1- and Dic1-dependent delivery of citrate and succinate (respectively) to mitochondria) (Supplementary Figure 2); 7) it increases the concentrations of Mpc1 and Mpc3, the two protein components of a mitochondrial pyruvate carrier involved in pyruvate transport to mitochondria during respiratory growth;it does not, however, alter the concentration of the Mpc2 protein component of the mitochondrial pyruvate carrier Mpc1/Mpc2 that assembles and operates during fermentative growth (Supplementary Figure 2); 8) it increases the percentage of cells exhibiting a tubular mitochondrial network and decreases the percentage of cells displaying fragmented mitochondria; 9) it lowers cell susceptibility to an apoptotic mode of regulated cell death (RCD) caused by an exposure to hydrogen peroxide or acetic acid; and 9) it decreases cell susceptibility to liponecrotic RCD triggered by a treatment with FFA [36, 44, 50, 52–54, 72]. It remained unclear how LCA regulates the anabolic and catabolic branches of TAG metabolism in the ER, LD and peroxisomes (Supplementary Figure 1), and how it regulates other cellular processes named in this section. It was also unknown if and how such LCA-dependent regulation of some (or all) of these cellular processes contributes to the ability of LCA to delay yeast chronological aging. We put forward the hypothesis that LCA may delay chronological aging of yeast under CR also because this bile acid affects these other cellular processes confined to different cellular locations. To test this hypothesis, we examined mechanisms underlying the ability of LCA to regulate the spatiotemporal dynamics of these other cellular processes confined to several compartments of yeast cells limited in calorie supply. We also assessed a possibility that there is a causal relationship between such LCA-driven regulation of these other cellular processes and the LCA-dependent delay of yeast chronological aging under CR conditions.

### LCA increases the abundance of LD in an age-related manner

**Figure 1:**
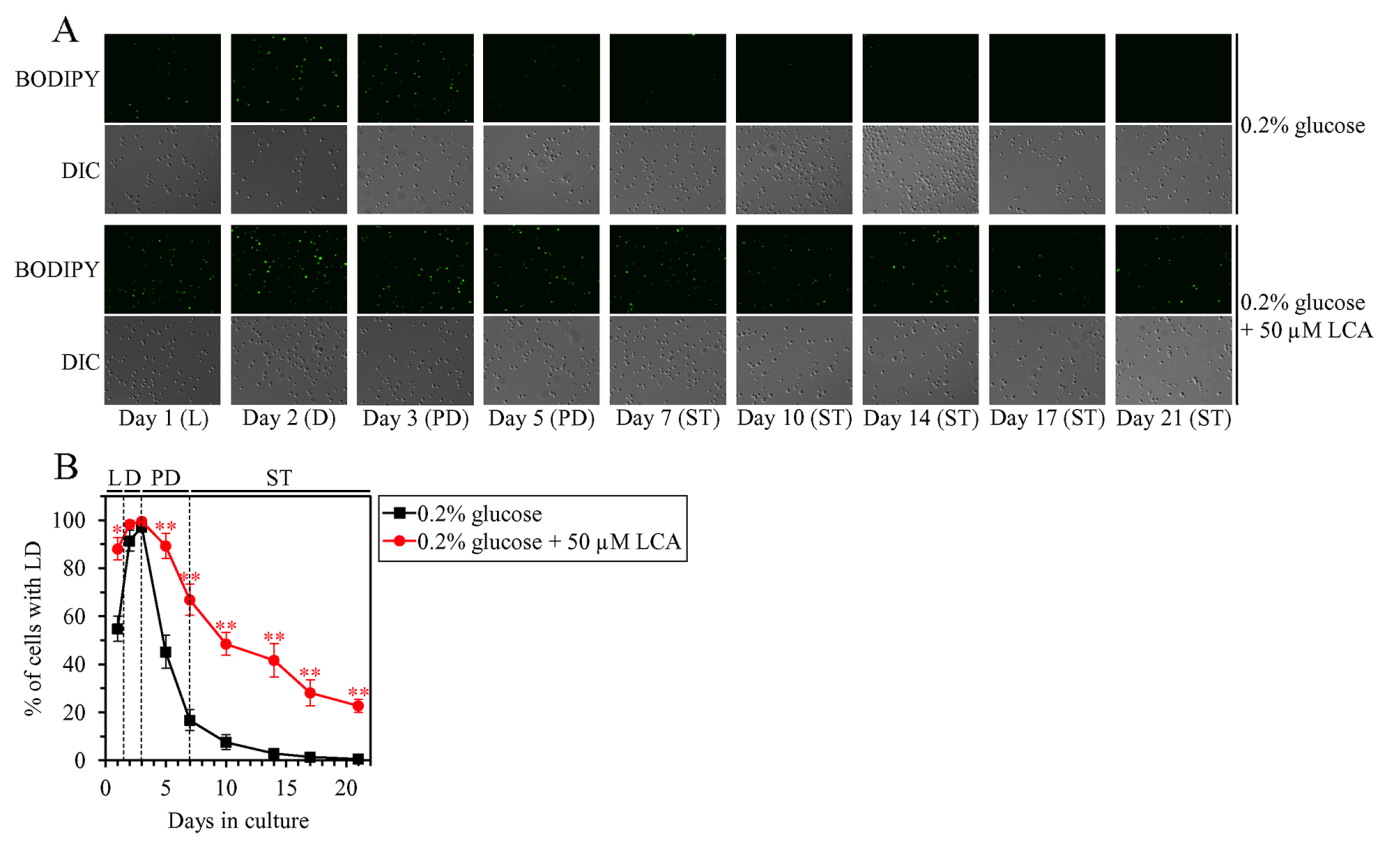
Under CR conditions, LCA causes an age-related increase in the percentage of wild-type (WT) cells that contain lipid droplets (LD) WT cells were cultured in the nutrient-rich YP medium initially containing 0.2% glucose with 50 μM LCA or without it. **(A)** Cells recovered on different days of culturing were visualized using the differential interference contrast (DIC) microscopy and stained with BODIPY 493/503, which was used to detect neutral lipids of LD as described in Materials and Methods. **(B)** Percentage of cells that contain LD. Images like the representative images shown in (A) were quantitated. Data are presented as means ± SEM (n = 3; ^*^p < 0.05; ^**^p < 0.01). Abbreviations: L, D, PD and ST, logarithmic, diauxic, post-diauxic and stationary growth phases (respectively).

We have previously found that, during PD and ST phases of culturing yeast under CR on 0.2% glucose, LCA rises the abundance of TAG and increases the concentrations of enzymes involved in the synthesis of this class of neutral lipids from FFA in the ER [36, 50, 53]. We have also noticed that, in these yeast cells, LCA decreases the abundance of FFA and decreases the concentrations of enzymes involved in the lipolytic conversion of TAG into FFA in LD [36, 50, 53]. Based on the above observations, we hypothesized that LCA may have the following effects on TAG metabolism and transport in yeast limited in calorie supply: 1) it may accelerate TAG synthesis in the ER and, perhaps, the ensuing TAG deposition in LD; and 2) it may decelerate TAG lipolysis in LD. This hypothesis posits that LCA may increase the abundance of LD in chronologically aging yeast under CR conditions, perhaps during certain stages of the aging process (i.e. in an age-related manner). To test this hypothesis, we used live-cell fluorescence microscopy with BODIPY 493/503, a stain for neutral lipids, to assess the percentage of cells that contain LD in wild-type (WT) strain cultured under CR conditions with or without LCA; the cells were recovered on different days (i.e. in different phases) of culturing. We found that LCA has the following effects: 1) it increases the percentage of cells with LD in logarithmic (L) phase (Figure 1A and 1B), when neutral lipids are known to be synthesized in the ER and deposited in LD [73]; and 2) it rises the percentage of cells with LD in PD and ST phases (Figure 1A and 1B), when neutral lipids are known to be lipolytically degraded in LD [73].

These findings support our hypothesis on the following effects of LCA in yeast cells of WT strain limited in calorie supply: 1) LCA accelerates TAG synthesis in the ER and the subsequent TAG deposition in LD; and 2) LCA decelerates TAG lipolysis in LD. The first effect of LCA prevails during L phase of culturing under CR conditions, whereas the second effect of LCA predominates during PD and ST phases of such culturing.

### LCA decreases the concentration of FFA during several consecutive stages of the aging process

**Figure 2:**
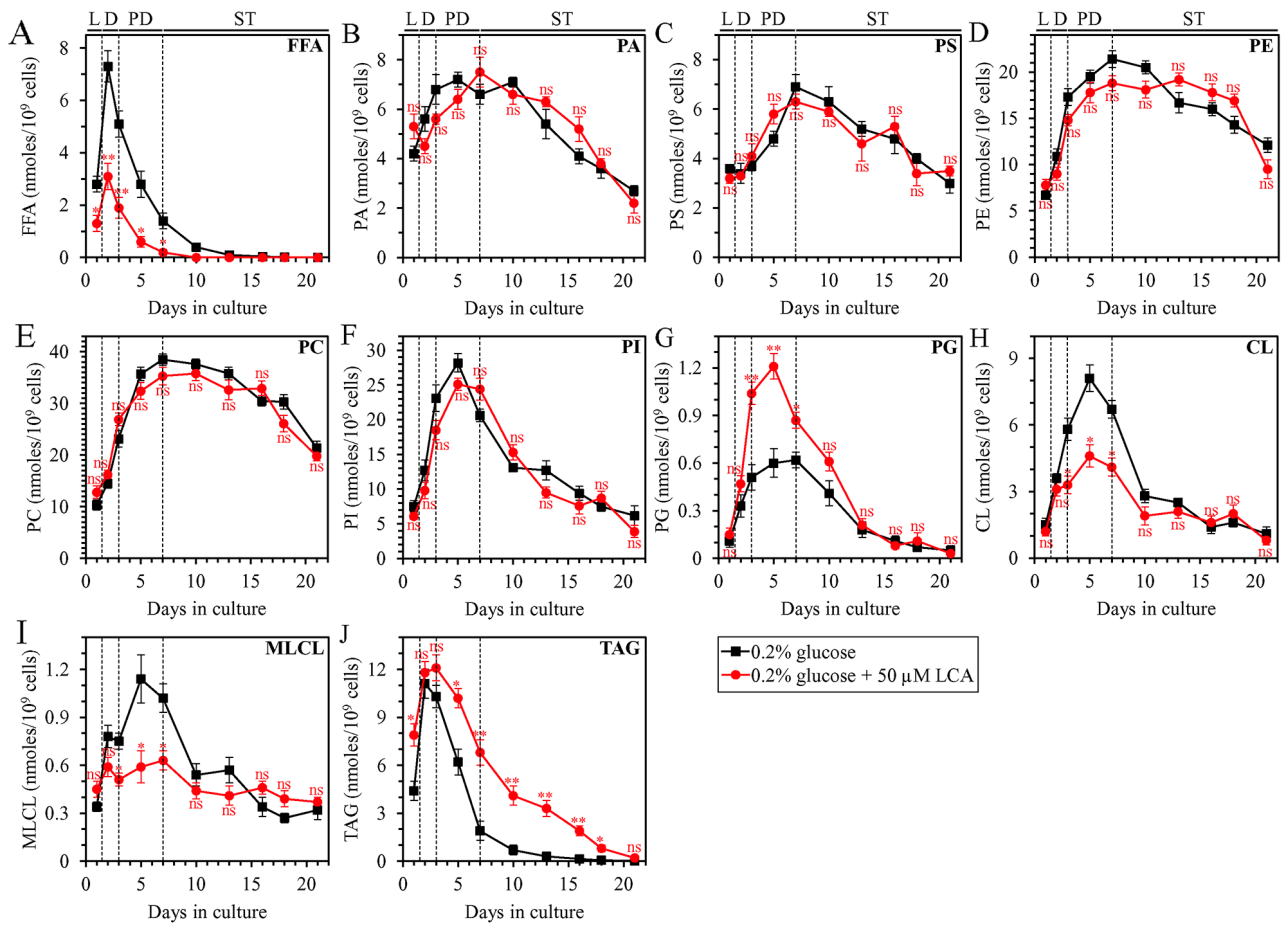
Under CR conditions, LCA exhibits age-related differential effects on the concentrations of several classes of cellular lipids in WT yeast WT cells were cultured in the nutrient-rich YP medium initially containing 0.2% glucose with 50 μM LCA or without it. Following extraction of lipids from cells recovered on different days of culturing, various lipid classes were identified and quantitated by mass spectrometry as described in Materials and Methods. These lipid classes included free fatty acids (FFA) **(A)**, phosphatidic acid (PA) **(B)**, phosphatidylserine (PS) **(C)**, phosphatidylethanolamine (PE) **(D)**, phosphatidylcholine (PC) **(E)**, phosphatidylinositol (PI) **(F)**, phosphatidylglycerol (PG) **(G)**, cardiolipin (CL) **(H)**, monolysocardiolipin (monolysocardiolipin) **(I)** and triacylglycerol (TAG) **(J)**. Data are presented as means ± SEM (n = 3; ^*^p < 0.05; ^**^p < 0.01; ns, not significant). Abbreviations: CL, cardiolipin; FFA, free fatty acids; L, D, PD and ST, logarithmic, diauxic, post-diauxic and stationary growth phases (respectively); MLCL, monolysocardiolipin; PA, phosphatidic acid; PC, phosphatidylcholine; PE, phosphatidylethanolamine; PG, phosphatidylglycerol; PI, phosphatidylinositol; PS, phosphatidylserine; TAG, triacylglycerol.

As we found, LCA accelerates TAG synthesis from FFA during L phase and decelerates TAG lipolysis into FFA during PD and ST phases of culturing under CR conditions. We therefore hypothesized that LCA may decrease the concentration of FFA through several phases of culturing under CR conditions. To test this hypothesis, we used quantitative mass spectrometry to compare cellular lipidomes of WT yeast cultured under CR conditions with or without LCA. In support of our hypothesis, we found that LCA causes a significant decline in the concentration of FFA in L, diauxic (D), PD and the beginning of ST phases (Figure 2A).

Furthermore, almost mirroring the effects of LCA on the temporal dynamics of TAG-containing LD (Figure 1B), LCA affected the intracellular concentration of TAG as follows: 1) LCA increased TAG concentration in L phase; 2) LCA had no significant effect on TAG concentration in D phase; and 3) LCA increased TAG concentration in PD and ST phases (Figure 2J).

Moreover, as we have described previously [45, 52–54], because within a yeast cell LCA amasses primarily in both mitochondrial membranes and specifically alters phospholipid metabolism and transport in these membranes, LCA exhibited the following effects on the cellular concentrations of the signature mitochondrial membrane lipids cardiolipin (CL), monolysocardiolipin (MLCL) and phosphatidylglycerol (PG): 1) in D and PD phases, LCA decreased the concentrations of CL and MLCL, both of which are synthesized in the IMM from PG (Figures 2H and 2I, respectively); and 2) in D and PD phases, LCA also increased the concentration of PG (Figure 2G).

Unlike the ability of LCA to alter the cellular concentrations of FFA, TAG, CL, MLCL and PG, this bile acid had no significant effect on the concentrations of the following phospholipids: phosphatidic acid (PA), phosphatidylserine (PS), phosphatidylethanolamine (PE), phosphatidylcholine (PC) and phosphatidylinositol (PI) (Figures 2B, 2C, 2D, 2E and 2F, respectively).

In sum, these findings further support our hypothesis on the mechanism through which LCA regulates the anabolic branch of TAG metabolism in the ER and the catabolic branch of TAG metabolism in LD. In this mechanism: 1) by accelerating TAG synthesis from FFA within the ER and the subsequent TAG deposition within LD during L and, likely, D phases of culturing under CR conditions, LCA decreases the concentration of FFA during these two phases; and 2) by decelerating TAG lipolysis into FFA within LD during PD and ST phases of culturing under CR conditions, LCA decreases the concentration of FFA during these two phases (Supplementary Figure 3).

### The efficiency of longevity extension by LCA inversely correlates with the intracellular concentration of FFA

Because LCA causes a significant decline in the concentration of FFA during several consecutive phases of culturing under CR conditions, we sought to determine if the extent of such decline correlates with the efficiency of longevity extension by LCA under these conditions.

**Figure 3:**
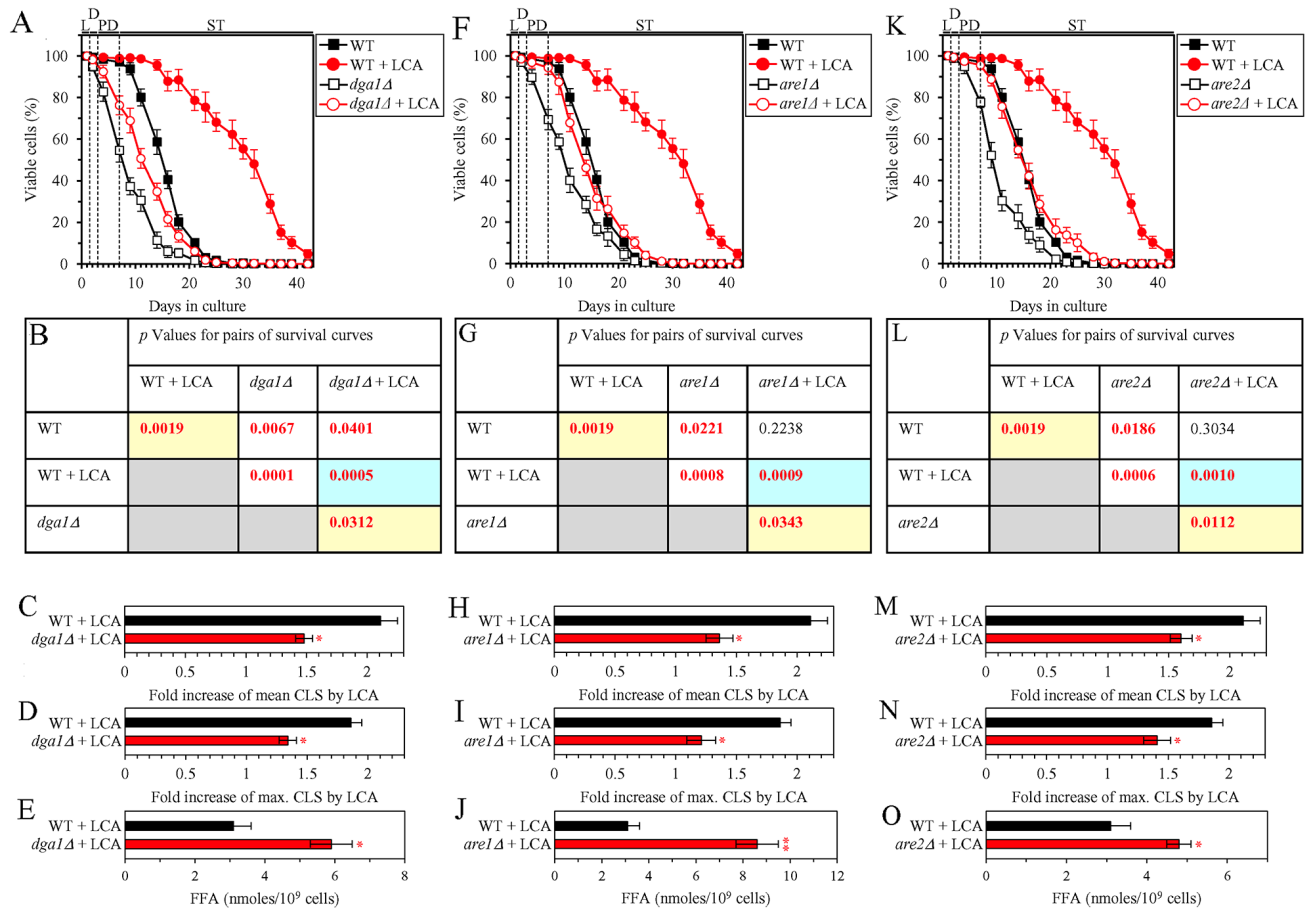
Under CR conditions in the presence of LCA, lack of any of the three enzymes involved in the synthesis of TAG from FFA increases the concentration of FFA and decreases the extent to which LCA can extend yeast chronological lifespan (CLS) WT cells and mutant cells carrying a single-gene-deletion mutation eliminating either the Dga1, Are1 or Are2 protein were cultured in the nutrient-rich YP medium initially containing 0.2% glucose with 50 μM LCA or without it. **(A, F, K)** Survival curves of the chronologically aging WT and *dga1Δ* (A), WT and *are1Δ* (F) or WT and *are2Δ* (K) strains are shown. Data are presented as means ± SEM (n = 3). Data for the WT strain cultured with or without LCA are replicated in graphs A, F, K of this Figure. **(B, G, L)** *p* Values for different pairs of survival curves of the WT and *dga1Δ* (B), WT and *are1Δ* (G) or WT and *are2Δ* (L) strains cultured with or without LCA. Survival curves shown in (A, F or K, respectively) were compared. Two survival curves were considered statistically different if the *p* value was less than 0.05. The *p* values for comparing pairs of survival curves using the logrank test were calculated as described in Materials and Methods. The *p* values displayed on a yellow color background indicate that LCA extends the CLS of the WT, *dga1Δ* (B), *are1Δ* (G) and *are2Δ* (L) strains. The *p* values displayed on a blue color background indicate that LCA extends the CLS of the *dga1Δ* (B), *are1Δ* (G) and *are2Δ* (L) strains to a lower extent than that of the WT strain. **(C, D, H, I, M, N)** Survival curves shown in (A, F, K) were used to calculate the fold of increase of the mean (C, H, M) and maximum (D, I, N) CLS by LCA for the WT and *dga1Δ* (C, D), WT and *are1Δ* (H, I) or WT and *are2Δ* (M, N) strains. Data are presented as means ± SEM (n = 3; ^*^p < 0.05). **(E, J, O)** The maximum concentration of FFA, which was observed in WT and *dga1Δ* (E), WT and *are1Δ* (J) or WT and *are2Δ* (O) cells recovered on day 2 of culturing with LCA, is shown. Data are presented as means ± SEM (n = 3; ^*^p < 0.05; ^**^p < 0.01). Abbreviations: FFA, free fatty acids; L, D, PD and ST, logarithmic, diauxic, post-diauxic and stationary growth phases (respectively).

We first assessed how a single-gene-deletion mutation eliminating the Dga1, Are1 or Are2 protein, each catalyzing the synthesis of TAG from FFA in the ER (Supplementary Figure 3), influences the efficiency of yeast chronological lifespan (CLS) extension by LCA and how it affects the cellular concentration of FFA under CR conditions. We found that the *dga1Δ*, *are1Δ* and *are2Δ* mutations exhibit the following effects: 1) each of them significantly decreases the extent to which LCA can extend both the mean and maximum CLS (Figures 3A-3D for *dga1Δ*, Figures 3F-3I for *are1Δ* and Figures 3K-3N for *are2Δ*); and 2) each of them significantly increases the cellular concentration of FFA (Figures 3E, 3J and 3O for *dga1Δ*, *are1Δ* and *are2Δ* [respectively]). Using these data, we compared the fold increase of mean or maximum CLS and the maximum intracellular concentration of FFA (which was observed in WT, *dga1Δ*, *are1Δ* and *are2Δ* cells recovered on day 2 of culturing with LCA under CR conditions). We found that the Pearson’s correlation coefficient (r) values for the correlation between these two compared variables are less than -0.8 for both possible pairwise combinations of the mean or maximum CLS and the maximum intracellular concentration of FFA (Supplementary Figure 4). Because the Pearson’s r value ranging from -0.7 to -0.9 is considered a high negative correlation between the two variables [74], we concluded that the fold increase of mean or maximum CLS has a high negative correlation with the intracellular concentration of FFA. Thus, the efficiency of longevity extension by LCA inversely correlates with the intracellular concentration of FFA.

**Figure 4:**
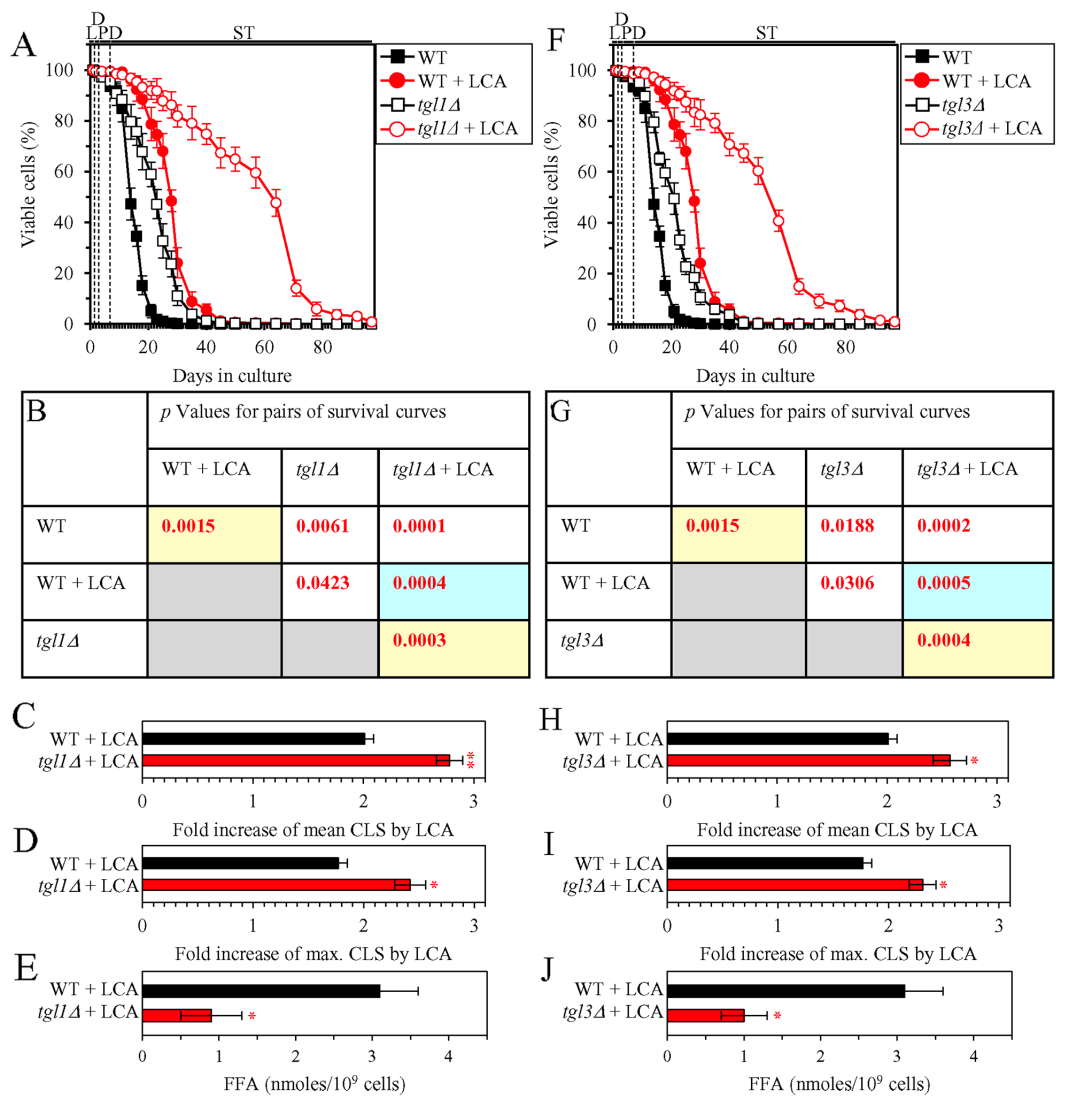
Under CR conditions in the presence of LCA, lack of the Tgl1 or Tgl3 enzymes involved in the TAG lipolysis that yields FFA decreases the concentration of FFA and increases the extent to which LCA can extend yeast CLS WT cells and mutant cells carrying a single-gene-deletion mutation eliminating either the Tgl1 or Tgl3 protein were cultured in the nutrient-rich YP medium initially containing 0.2% glucose with 50 μM LCA or without it. **(A, F)** Survival curves of the chronologically aging WT and *tgl1Δ* (A) or WT and *tgl3Δ* (F) strains are shown. Data are presented as means ± SEM (n = 3). Data for the WT strain cultured with or without LCA are replicated in graphs A, F of this Figure and in graphs A, F of Figure 5. **(B, G)** *p* Values for different pairs of survival curves of the WT and *tgl1Δ* (B) or WT and *tgl3Δ* (G) strains cultured with or without LCA. Survival curves shown in A or F (respectively) were compared. Two survival curves were considered statistically different if the *p* value was less than 0.05. The *p* values for comparing pairs of survival curves using the logrank test were calculated as described in Materials and Methods. The *p* values displayed on a yellow color background indicate that LCA extends the CLS of the WT (B and G), *tgl1Δ* (B) and *tgl3Δ* (G) strains. The *p* values displayed on a blue color background indicate that LCA extends the CLS of the *tgl1Δ* (B) and *tgl3Δ* (G) strains to a higher extent that that of the WT strain. **(C, D, H, I)** Survival curves shown in (A, F) were used to calculate the fold of increase of the mean (C, H) and maximum (D, I) CLS by LCA for the WT and *tgl1Δ* (C, D) or WT and *tgl3Δ* (H, I) strains. Data are presented as means ± SEM (n = 3; ^*^p < 0.05; ^**^p < 0.01). **(E, J)** The maximum concentration of FFA, which was observed in WT and *tgl1Δ* (E) or WT and *tgl3Δ* (J) cells recovered on day 2 of culturing with LCA, is shown. Data are presented as means ± SEM (n = 3; ^*^p < 0.05). Abbreviations: FFA, free fatty acids; L, D, PD and ST, logarithmic, diauxic, post-diauxic and stationary growth phases (respectively).

**Figure 5:**
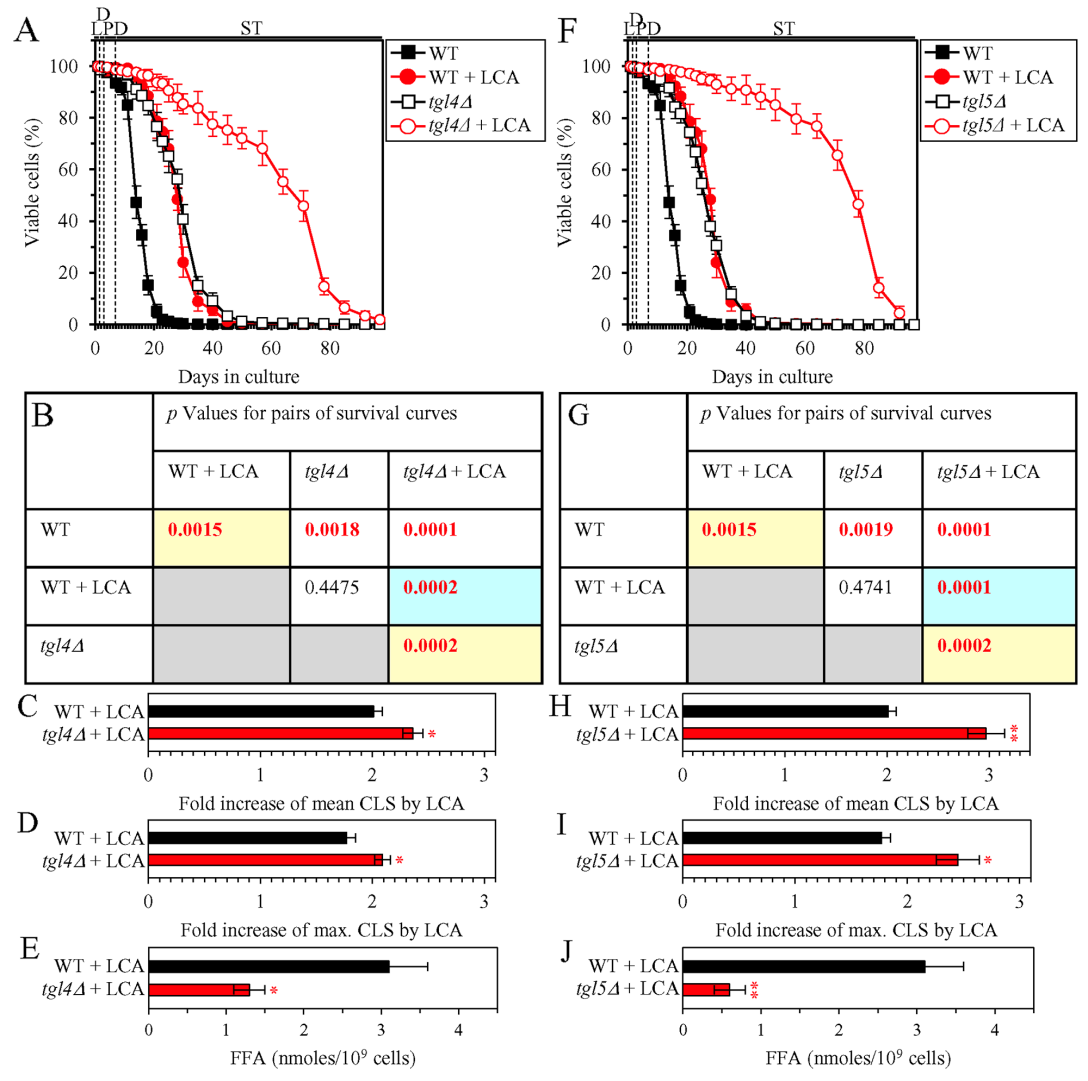
Under CR conditions in the presence of LCA, lack of the Tgl4 or Tgl5 enzymes involved in the TAG lipolysis that yields FFA decreases the concentration of FFA and increases the extent to which LCA can extend yeast CLS WT cells and mutant cells carrying a single-gene-deletion mutation eliminating either the Tgl4 or Tgl5 protein were cultured in the nutrient-rich YP medium initially containing 0.2% glucose with 50 μM LCA or without it. **(A, F)** Survival curves of the chronologically aging WT and *tgl4Δ* (A) or WT and *tgl5Δ* (F) strains are shown. Data are presented as means ± SEM (n = 3). Data for the WT strain cultured with or without LCA are replicated in graphs A, F of this Figure and in graphs A, F of Figure 4. **(B, G)** *p* Values for different pairs of survival curves of the WT and *tgl4Δ* (B) or WT and *tgl5Δ* (G) strains cultured with or without LCA. Survival curves shown in A or F (respectively) were compared. Two survival curves were considered statistically different if the *p* value was less than 0.05. The *p* values for comparing pairs of survival curves using the logrank test were calculated as described in Materials and Methods. The *p* values displayed on a yellow color background indicate that LCA extends the CLS of the WT (B and G), *tgl4Δ* (B) and *tgl5Δ* (G) strains. The *p* values displayed on a blue color background indicate that LCA extends the CLS of *tgl4Δ* (B) and *tgl5Δ* (G) strains to a higher extent that that of the WT strain. **(C, D, H, I)** Survival curves shown in (A, F) were used to calculate the fold of increase of the mean (C, H) and maximum (D, I) CLS by LCA for the WT and *tgl4Δ* (C, D) or WT and *tgl5Δ* (H, I) strains. Data are presented as means ± SEM (n = 3; ^*^p < 0.05; ^**^p < 0.01). **(E, J)** The maximum concentration of FFA, which was observed in WT and *tgl4Δ* (E) or WT and *tgl5Δ* (J) cells recovered on day 2 of culturing with LCA, is shown. Data are presented as means ± SEM (n = 3; ^*^p < 0.05; ^**^p <0.01). Abbreviations: FFA, free fatty acids; L, D, PD and ST, logarithmic, diauxic, post-diauxic and stationary growth phases (respectively).

We then investigated how a single-gene-deletion mutation eliminating the Tgl1, Tgl3, Tgl4 or Tgl5 protein, each catalyzing TAG lipolysis that yields FFA in LD (Supplementary Figure 3), affects the efficiency of yeast CLS extension by LCA and how it influences the cellular concentration of FFA under CR conditions. We found that the *tgl1Δ*, *tgl3Δ*, *tgl4Δ* and *tgl5Δ* mutations have the following effects: 1) each of them significantly increases the extent to which LCA can prolong both the mean and maximum CLS (Figures 4A-4D for *tgl1Δ*, Figures 4F-4I for *tgl3Δ*, Figures 5A-5D for *tgl4Δ* and Figures 5F-5I for *tgl5Δ*); and 2) each of them significantly decreases the cellular concentration of FFA (Figures 4E, 4J, 5E and 5J for *tgl1Δ*, *tgl3Δ*, *tgl4Δ* and *tgl5Δ* [respectively]). We used these data to compare the fold increase of mean or maximum CLS and the maximum intracellular concentration of FFA (which was observed in WT, *tgl1Δ*, *tgl3Δ*, *tgl4Δ* and *tgl5Δ* cells recovered on day 2 of culturing with LCA under CR conditions). The Pearson’s r values for the correlation between these two variables were less than -0.9 for both possible pairwise combinations (Supplementary Figure 5). Hence, the fold increase of mean or maximum CLS has a very high negative correlation with the intracellular concentration of FFA. These results confirm our assumption that the efficiency of longevity extension by LCA inversely correlates with the intracellular concentration of FFA.

In sum, the above findings indicate that LCA delays yeast chronological aging in part because it decreases the intracellular concentration of FFA.

### LCA delays the age-related onset and decelerates progression of liponecrosis and decreases cell susceptibility to this form of RCD in an age-dependent manner

Our recent study has revealed that CR extends yeast CLS in part because this low-calorie diet allows cells to maintain a low concentration of FFA during PD and ST phases, thereby postponing the onset of the liponecrotic mode of RCD [75]. The present study indicates that in yeast cultured under CR conditions, LCA causes further decline in the concentration of FFA in L, D, PD and the beginning of ST phases (Figure 2A). We therefore sought to determine if LCA may further postpone the age-related onset of liponecrotic RCD in chronologically aging yeast under CR conditions.

**Figure 6:**
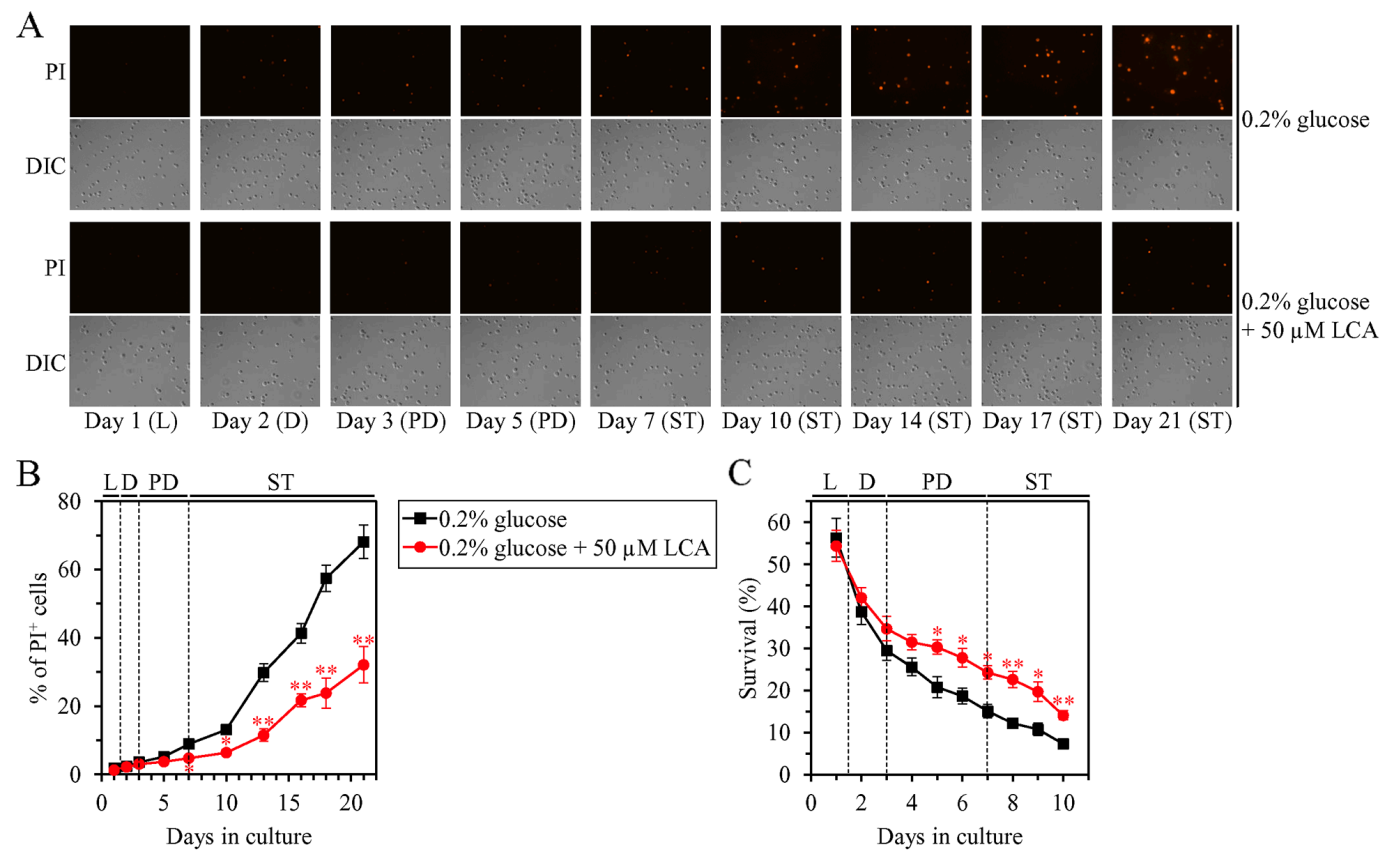
Under CR conditions, LCA postpones the age-related onset of necrotic cell death and decreases cell susceptibility to a liponecrotic mode of regulated cell death (RCD) WT cells were cultured in the nutrient-rich YP medium initially containing 0.2% glucose with 50 μM LCA or without it. **(A)** Cells recovered on different days of culturing were visualized using the DIC microscopy and stained with propidium iodide (PI) for visualizing the loss of plasma membrane integrity (a hallmark event of necrosis) as described in Materials and Methods. **(B)** Percentage of cells exhibiting PI positive staining characteristic of necrotic cell death. Images like the representative images shown in (A) were quantitated. Data are presented as means ± SEM (n = 3; ^*^p < 0.05; ^**^p < 0.01). Data for the WT strain cultured with LCA are replicated in graph A of Figure 7. **(C)** Clonogenic survival of cells recovered on different days of culturing and then treated for 2 h with 0.15 mM palmitoleic acid (POA, a monounsaturated form of FFA) to elicit a liponecrotic mode of RCD as described in Materials and Methods. Data are presented as means ± SEM (n = 3; ^*^p < 0.05; ^**^p < 0.01). Data for the WT strain cultured with LCA are replicated in graph D of Figure 7. Abbreviations: L, D, PD and ST, logarithmic, diauxic, post-diauxic and stationary growth phases (respectively).

We first used live-cell fluorescence microscopy with propidium iodide (PI), a stain for visualizing the loss of plasma membrane (PM) integrity (a hallmark event of necrosis), to monitor how LCA influences age-related changes in the percentage of calorically restricted WT cells exhibiting PI positive staining; this staining is characteristic of necrotic cell death. We found that in WT cells LCA delays the onset of necrosis in the end of PD phase and slows down the progression of this cell death mode during ST phase of culturing under CR conditions (Figure 6A and 6B).

We then examined if LCA influences the susceptibility of calorically restricted WT cells to liponecrotic RCD; this mode of age-related RCD can be triggered by a short-term treatment of yeast with FFA [36, 76–79]. We found that in WT yeast cultured under CR conditions, LCA significantly increases clonogenic survival of cells briefly (for 2 h) treated with palmitoleic acid (POA; a monounsaturated form of FFA) if these cells were recovered during PD or ST phase of culturing (Figure 6C).

These findings indicate that in WT yeast limited in calorie supply, LCA postpones the age-related onset and slows down the progression of liponecrotic RCD and decreases cell susceptibility to this mode of RCD in an age-dependent manner. It is conceivable that LCA may extend longevity of chronologically aging WT cells under CR conditions in part by decreasing the risk of liponecrotic RCD during PD and ST phases and actively increasing the chance of cell survival during these phases.

### The efficiency with which LCA decreases the risk of age-related liponecrotic RCD inversely correlates with the intracellular concentration of FFA

Since LCA elicits a substantial reduction in the concentration of FFA in yeast cultured under CR conditions, we assessed if the extent of such reduction correlates with the efficiencies of the following LCA-dependent processes: 1) the delay of the age-related onset of liponecrotic RCD; 2) the deceleration of progression of liponecrotic RCD; and 3) the decrease in cell susceptibility to liponecrotic RCD.

**Figure 7:**
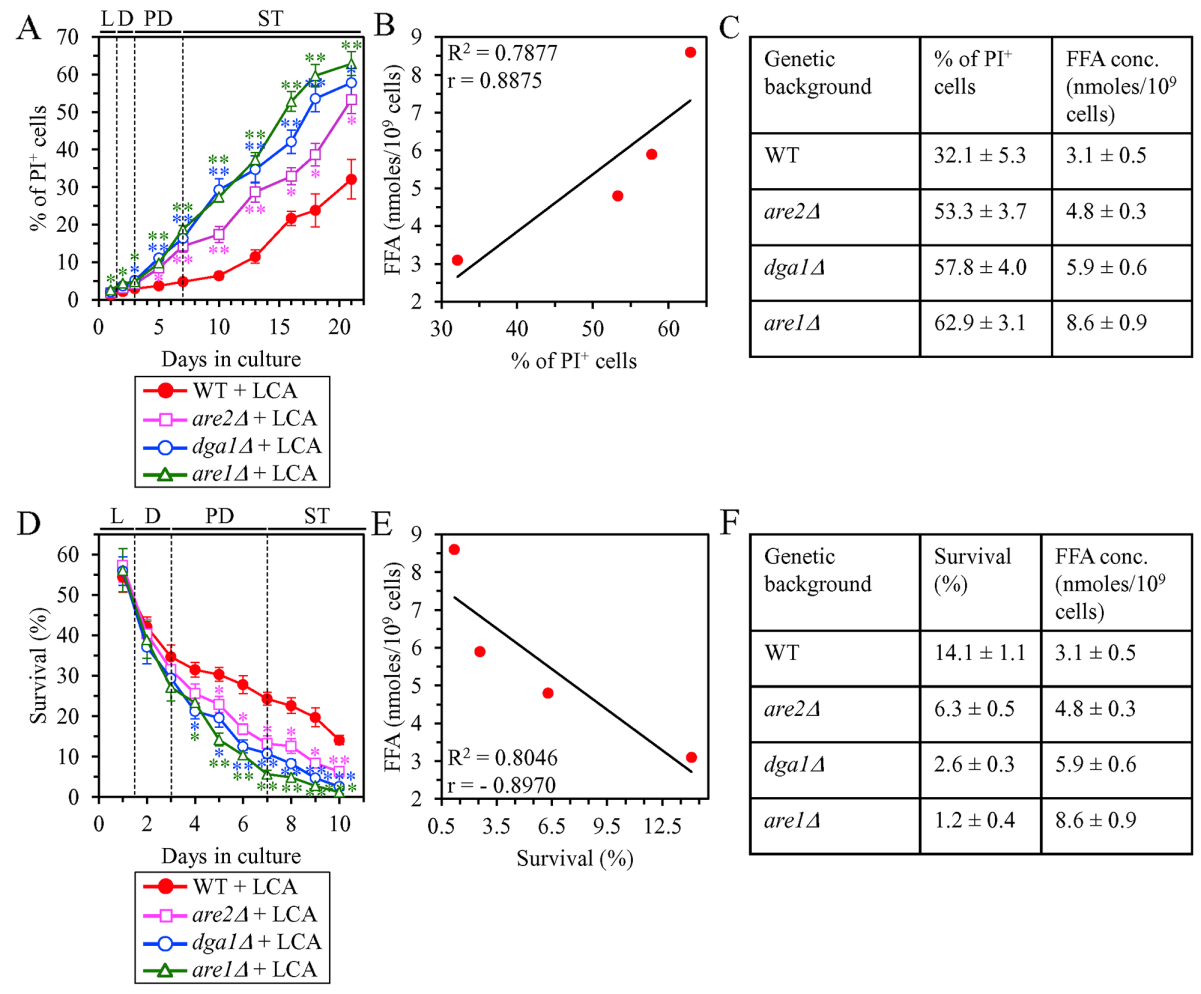
Under CR conditions in the presence of LCA, lack of any of the three enzymes involved in the synthesis of TAG from FFA accelerates the age-related onset of necrotic cell death and increases cell susceptibility to liponecrotic RCD proportionally to the cellular concentration of FFA WT cells and mutant cells carrying a single-gene-deletion mutation eliminating either the Dga1, Are1 or Are2 protein were cultured in the nutrient-rich YP medium initially containing 0.2% glucose with 50 μM LCA. **(A)** Cells recovered on different days of culturing were visualized using the DIC microscopy and stained with PI for visualizing the loss of plasma membrane integrity (a hallmark event of necrosis) as described in Materials and Methods. Percentage of cells exhibiting PI positive staining characteristic of necrotic cell death is shown. Data are presented as means ± SEM (n = 3; ^*^p < 0.05; ^**^p < 0.01). Data for the WT strain cultured with LCA are replicated in graph B of Figure 6. **(B)** Plot comparing the maximum percentage of cells exhibiting PI positive staining (which was observed in WT and mutant cells recovered on day 21, the last day of culturing with LCA) and the maximum concentration of FFA (which was observed in WT and mutant cells recovered on day 2 of culturing with LCA). Different points show the data for WT, *dga1Δ*, *are1Δ* or *are2Δ* cells. Linear trendline and the R-squared value are displayed; the R-squared value demonstrates a good fit of the line to the data. The Pearson’s correlation coefficient (r) value is also shown; because the r value ranging from 0.7 to 0.9 is considered a high positive correlation between the two variables, the percentage of cells exhibiting PI positive staining has a high positive correlation with the intracellular concentration of FFA. **(C)** The experimental data used to create the plot shown in (B). Genetic backgrounds of strains, the percentage of cells exhibiting PI positive staining on day 21 (the last day) of culturing with LCA and the maximum concentration of FFA, which was observed in WT and mutant cells recovered on day 2 of culturing with LCA, are shown. Data are presented as means ± SEM (n = 3). **(D)** Clonogenic survival of cells recovered on different days of culturing and then treated for 2 h with 0.15 mM POA (a monounsaturated form of FFA) to elicit a liponecrotic mode of RCD as described in Materials and Methods. Data are presented as means ± SEM (n = 3; ^*^p < 0.05; ^**^p < 0.01). Data for the WT strain cultured with LCA are replicated in graph C of Figure 6. **(E)** Plot comparing the minimum percentage of clonogenic survival of POA-treated cells (which was observed in WT and mutant cells recovered on day 10, the last day of cell recovery from the culture for the treatment with POA) and the maximum concentration of FFA (which was observed in WT and mutant cells recovered on day 2 of culturing with LCA). Different points show the data for WT, *dga1Δ*, *are1Δ* or *are2Δ* cells. Linear trendline and the R-squared value are displayed; the R-squared value demonstrates a good fit of the line to the data. The Pearson’s correlation coefficient (r) value is also shown; because the r value ranging from -0.7 to -0.9 is considered a high negative correlation between the two variables, the percentage of clonogenic survival of POA-treated cells has a high negative correlation with the intracellular concentration of FFA. **(F)** The experimental data used to create the plot shown in (E). Genetic backgrounds of strains, the percentage of clonogenic survival of POA-treated cells on day 10 (the last day of cell recovery from the culture for the treatment with POA) and the maximum concentration of FFA, which was observed in WT and mutant cells recovered on day 2 of culturing with LCA, are shown. Data are presented as means ± SEM (n = 3). Abbreviations: FFA, free fatty acids; L, D, PD and ST, logarithmic, diauxic, post-diauxic and stationary growth phases (respectively); PI, propidium iodide.

We initially tested how a single-gene-deletion mutation eliminating the Dga1, Are1 or Are2 protein, each catalyzing the synthesis of TAG from FFA in the ER (Supplementary Figure 3), influences age-related changes in the following traits of yeast cells limited in calorie supply: 1) the percentage of cells that display PI positive staining characteristic of necrotic cell death; 2) the susceptibility of cells to liponecrotic RCD caused by a brief (for 2 h) treatment of these cells with POA; and 3) the intracellular concentration of FFA. We found that the *dga1Δ*, *are1Δ* and *are2Δ* mutations have the following effects: 1) since the middle of PD phase of cell culturing, each of them significantly increases the percentage of cells exhibiting PI positive staining typical of necrotic cell death (Figure 7A); 2) since the middle of PD phase of cell culturing, each of them significantly increases cell susceptibility to liponecrotic RCD caused by a 2-h treatment with POA (Figure 7D); 3) each of them significantly increases the cellular concentration of FFA (Figures 3E, 3J and 3O for *dga1Δ*, *are1Δ* and *are2Δ* [respectively]). We used these data to compare the maximum intracellular concentration of FFA (which was observed in WT, *dga1Δ*, *are1Δ* and *are2Δ* cells recovered on day 2 of culturing with LCA under CR conditions) as one variable and each of the following two variables: 1) the maximum percentages of cells exhibiting PI positive staining (which were observed in WT and mutant cells recovered on day 21, the last day of culturing with LCA); or 2) the minimum percentages of clonogenic survival of POA-treated cells (which were observed in WT and mutant cells recovered on day 10, the last day of cell recovery from the culture for the treatment with POA). The Pearson’s r value for the correlation between the maximum intracellular concentration of FFA and the maximum percentage of cells exhibiting PI positive staining was more than 0.8 (Figure 7B). Thus, the maximum percentage of cells exhibiting PI positive staining has a high positive correlation with the intracellular concentration of FFA. The Pearson’s r value for the correlation between the maximum intracellular concentration of FFA and the minimum percentage of clonogenic survival of POA-treated cells was less than -0.8 (Figure 7E). Hence, the minimum percentage of clonogenic survival of POA-treated cells has a high negative correlation with the intracellular concentration of FFA. We therefore concluded that there is an inverse relationship between the extent of the LCA-dependent decline in the concentration of FFA and the efficiencies of the following LCA-dependent processes: 1) the delay of the age-related onset of liponecrotic RCD; 2) the deceleration of progression of liponecrotic RCD; and 3) the decrease in cell susceptibility to liponecrotic RCD.

**Figure 8:**
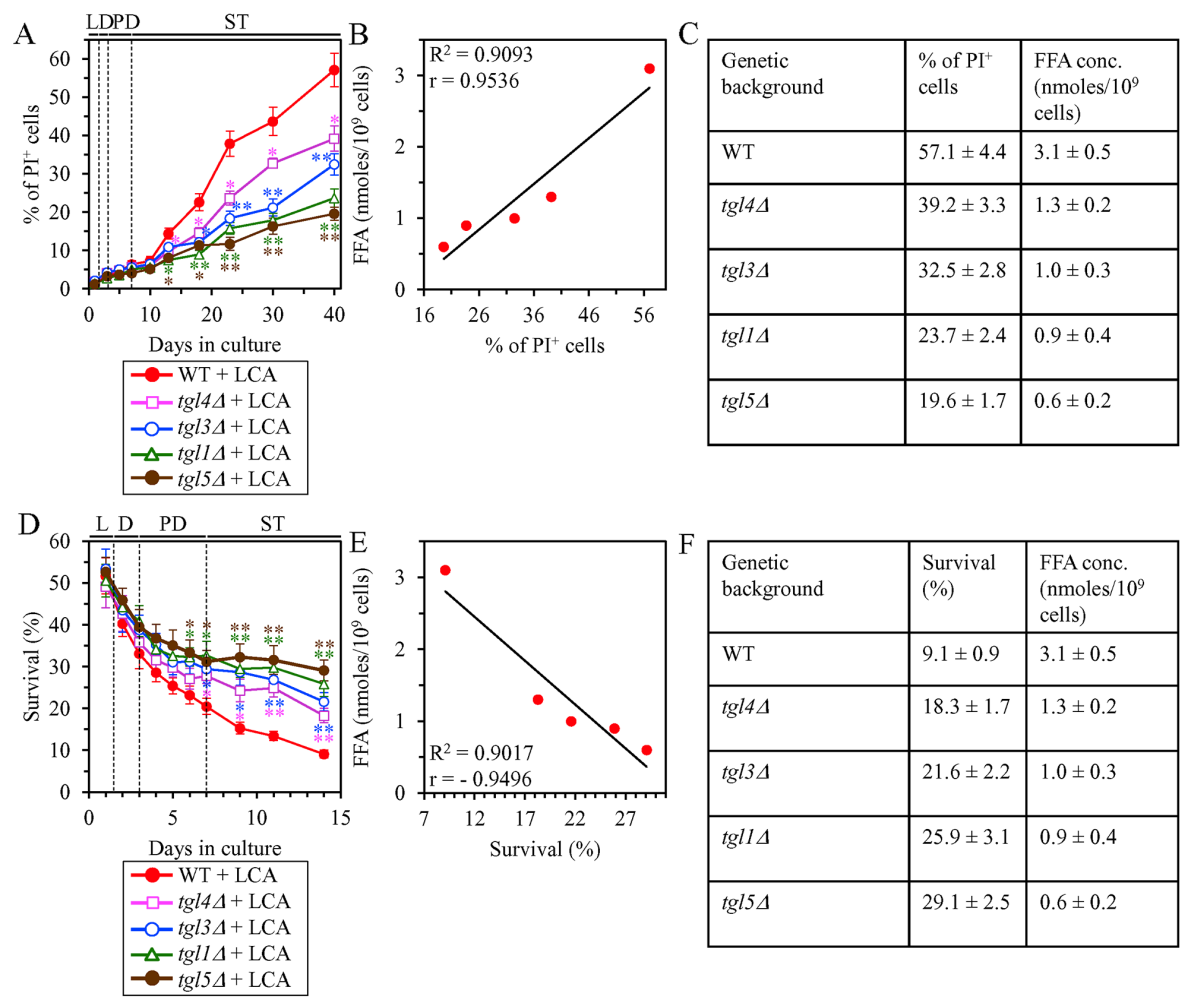
Under CR conditions in the presence of LCA, lack of any of the four enzymes involved in the TAG lipolysis that yields FFA decelerates the age-related onset of necrotic cell death and decreases cell susceptibility to liponecrotic RCD proportionally to the cellular concentration of FFA WT cells and mutant cells carrying a single-gene-deletion mutation eliminating either the Tgl1, Tgl3, Tgl4 or Tgl5 protein were cultured in the nutrient-rich YP medium initially containing 0.2% glucose with 50 μM LCA. **(A)** Cells recovered on different days of culturing were visualized using the DIC microscopy and stained with PI for visualizing the loss of plasma membrane integrity (a hallmark event of necrosis) as described in Materials and Methods. Percent of cells exhibiting PI positive staining characteristic of necrotic cell death is shown. Data are presented as means ± SEM (n = 3; ^*^p < 0.05; ^**^p < 0.01). **(B)** Plot comparing the maximum percentage of cells exhibiting PI positive staining (which was observed in WT and mutant cells recovered on day 40, the last day of culturing with LCA) and the maximum concentration of FFA (which was observed in WT and mutant cells recovered on day 2 of culturing with LCA). Different points show the data for WT, *tgl1Δ*, *tgl3Δ*, *tgl4Δ* or *tgl5Δ* cells. Linear trendline and the R-squared value are displayed; the R-squared value demonstrates a good fit of the line to the data. The Pearson’s correlation coefficient (r) value is also shown; because the r value ranging from 0.9 to 1.0 is considered a very high positive correlation between the two variables, the percentage of cells exhibiting PI positive staining has a very high positive correlation with the intracellular concentration of FFA. **(C)** The experimental data used to create the plot shown in (B). Genetic backgrounds of strains, the percentage of cells exhibiting PI positive staining on day 40 (the last day) of culturing with LCA and the maximum concentration of FFA, which was observed in WT and mutant cells recovered on day 2 of culturing with LCA, are shown. Data are presented as means ± SEM (n = 3). **(D)** Clonogenic survival of cells recovered on different days of culturing and then treated for 2 h with 0.15 mM POA (a monounsaturated form of FFA) to elicit a liponecrotic mode of RCD as described in Materials and Methods. Data are presented as means ± SEM (n = 3; ^*^p < 0.05; ^**^p < 0.01). **(E)** Plot comparing the minimum percentage of clonogenic survival of POA-treated cells (which was observed in WT and mutant cells recovered on day 14, the last day of POA treatment) and the maximum concentration of FFA (which were observed in WT and mutant cells recovered on day 2 of culturing with LCA). Different points show the data for WT, *tgl1Δ*, *tgl3Δ*, *tgl4Δ* or *tgl5Δ* cells. Linear trendline and the R-squared value are displayed; the R-squared value demonstrates a good fit of the line to the data. The Pearson’s correlation coefficient (r) value is also shown; because the r value ranging from -0.9 to -1.0 is considered a very high negative correlation between the two variables, the percentage of clonogenic survival of POA-treated cells has a very high negative correlation with the intracellular concentration of FFA. **(F)** The experimental data used to create the plot shown in (E). Genetic backgrounds of strains, the percentage of clonogenic survival of POA-treated cells on day 14 (the last day of POA treatment) and the maximum concentration of FFA, which was observed in WT and mutant cells recovered on day 2 of culturing with LCA, are shown. Data are presented as means ± SEM (n = 3). Abbreviations: FFA, free fatty acids; L, D, PD and ST, logarithmic, diauxic, post-diauxic and stationary growth phases (respectively); PI, propidium iodide.

We then investigated how a single-gene-deletion mutation eliminating the Tgl1, Tgl3, Tgl4 or Tgl5 protein, each catalyzing TAG lipolysis that yields FFA in LD (Supplementary Figure 3), affects age-related changes in the above traits of liponecrotic RCD and how it influences the cellular concentration of FFA under CR conditions. We found that the *tgl1Δ*, *tgl3Δ*, *tgl4Δ* and *tgl5Δ* mutations exhibit the following effects: 1) since the beginning of ST phase of cell culturing, each of them significantly decreases the the percentage of cells exhibiting PI positive staining characteristic of necrotic cell death (Figure 8A); 2) since the end of PD phase of cell culturing, each of them significantly decreases cell susceptibility to liponecrotic RCD elicited in response to a 2-h treatment with POA (Figure 8D); 3) each of them significantly decreases the cellular concentration of FFA (Figures 4E, 4J, 5E and 5J for *tgl1Δ*, *tgl3Δ*, *tgl4Δ* and *tgl5Δ* [respectively]). Using these data, we compared the maximum intracellular concentration of FFA (which was observed in WT, *tgl1Δ*, *tgl3Δ*, *tgl4Δ* and *tgl5Δ* cells recovered on day 2 of culturing with LCA under CR conditions) as one variable and each of the above traits of liponecrotic RCD as another variable. The Pearson’s r value for the correlation between the maximum intracellular concentration of FFA and the maximum percentage of cells exhibiting PI positive staining was more than 0.9 (Figure 8B). Hence, the maximum percentage of cells exhibiting PI positive staining has a very high positive correlation with the intracellular concentration of FFA. The Pearson’s r value for the correlation between the maximum intracellular concentration of FFA and the minimum percentage of clonogenic survival of POA-treated cells was less than -0.9 (Figure 8E). Thus, the minimum percentage of clonogenic survival of POA-treated cells has a very high negative correlation with the intracellular concentration of FFA. These results confirm our assumption that the extent of the LCA-dependent decline in the concentration of FFA is in an inverse relationship with the LCA-dependent postponement of the age-related onset of liponecrotic RCD, the LCA-dependent slowing of progression of liponecrotic RCD and the LCA-dependent decline in cell susceptibility to liponecrotic RCD.

It needs to be noted that the age-related onset of liponecrotic RCD and the rate of its progression in the above experiments were monitored in the absence of endogenous POA (a monounsaturated form of FFA), whereas cell susceptibility to liponecrotic RCD in these experiments was measured in yeast exposed to exogenous POA. Our previous studies have revealed that the buildup of POA-containing phospholipids in the PM and the excessive accumulation of POA-containing phospholipids in both mitochondrial membranes are direct pro-death processes in yeast treated with POA, whereas the assimilation of POA into TAG in the ER and the ensuing buildup of POA-containing TAG in LD are direct pro-survival processes in this yeast [76–79]. We have also previously demonstrated that the *dga1Δ* and *are2Δ* mutations, both of which decelerate the synthesis of TAG from FFA in the ER (Supplementary Figure 3) and decrease the incorporation of POA into TAG, increase cell susceptibility to liponecrotic RCD [76, 77]. These previous findings indicate the following: 1) an increase in the intracellular concentration of POA (a 16-carbon monounsaturated form of FFA) and the subsequent incorporation of POA into phospholipids increase the risk of liponecrotic RCD; and 2) a decrease in the intracellular concentration of POA caused by the assimilation of this form of FFA into TAG decreases the risk of liponecrotic RCD.

Altogether, the above findings and our previously published data [76–79] indicate that the efficiency of decreasing the risk of age-related liponecrotic RCD by LCA inversely correlates with the intracellular concentration of FFA. Thus, in support of our hypothesis proposed at the end of the previous section, LCA delays yeast chronological aging under CR conditions in part by decreasing the risk of liponecrotic RCD during PD and ST phases and actively increasing the chance of cell survival during these phases.

### The peroxisome-to-mitochondrion transport of acetyl-CoA via the carnitine shuttle is essential for the delay of yeast chronological aging by LCA

**Figure 9:**
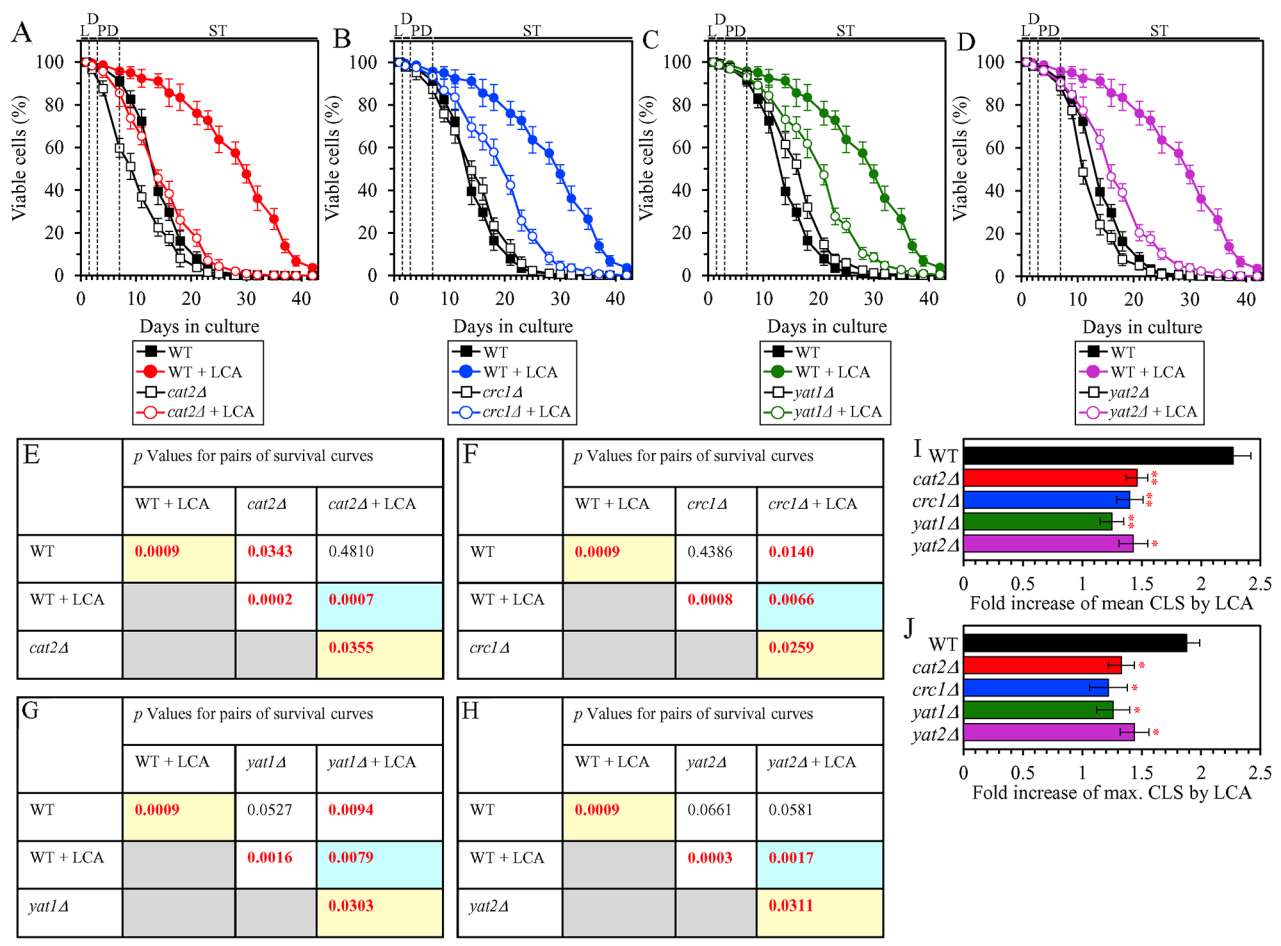
Under CR conditions in the presence of LCA, lack of any of the four proteins required for the transport of acetyl-CoA from peroxisomes to mitochondria via the carnitine shuttle decreases the efficiency of yeast CLS extension by LCA WT cells and mutant cells carrying a single-gene-deletion mutation eliminating either the Cat2, Crc1, Yat1 or Yat2 protein were cultured in the nutrient-rich YP medium initially containing 0.2% glucose with 50 μM LCA or without it. **(A, B, C, D)** Survival curves of the chronologically aging WT and *cat2Δ* (A), WT and *crc1Δ* (B), WT and *yat1Δ* (C) or WT and *yat2Δ* (D) strains are shown. Data are presented as means ± SEM (n = 3). Data for the WT strain cultured with or without LCA are replicated in graphs A, B, C, D of this Figure. **(E, F, G, H)** *p* Values for different pairs of survival curves of the WT and *cat2Δ* (E), WT and *crc1Δ* (F), WT and *yat1Δ* (G) or WT and *yat2Δ* (H) strains cultured with or without LCA. Survival curves shown in (A, B, C or D, respectively) were compared. Two survival curves were considered statistically different if the *p* value was less than 0.05. The *p* values for comparing pairs of survival curves using the logrank test were calculated as described in Materials and Methods. The *p* values displayed on a yellow color background indicate that LCA extends the CLS of the WT, *cat2Δ* (E), *crc1Δ* (F), *yat1Δ* (G) and *yat2Δ* (H) strains. The *p* values displayed on a blue color background indicate that LCA extends the CLS of the *cat2Δ* (E), *crc1Δ* (F), *yat1Δ* (G) and *yat2Δ* (H) strains to a lower extent than that of the WT strain. **(I, J)** Survival curves shown in (A, B, C, D) were used to calculate the fold of increase of the mean (I) and maximum (J) CLS by LCA for the WT, *cat2Δ*, *crc1Δ*, *yat1Δ* and *yat2Δ* strains. Data are presented as means ± SEM (n = 3; ^*^p < 0.05; ^**^p < 0.01). Abbreviations: L, D, PD and ST, logarithmic, diauxic, post-diauxic and stationary growth phases (respectively).

We have previously noticed that, during PD and ST phases of culturing yeast under CR on 0.2% glucose, LCA increases the concentrations of Cat2, Crc1, Yat1 and Yat2 [36, 50, 53]. All these proteins are known to be required for the carnitine-dependent transport of acetyl-CoA from peroxisomes (where acetyl-CoA is made as the final product of the β-oxidation of FFA) to mitochondria [80, 81] (Supplementary Figure 2). We hypothesized that such peroxisome-to-mitochondrion transport of acetyl-CoA via the carnitine shuttle may contribute to the ability of LCA to postpone chronological aging of yeast limited in calorie supply. To test this hypothesis, we investigated how a single-gene-deletion mutation eliminating the Cat2, Crc1, Yat1 or Yat2 protein influences the efficiency of yeast CLS extension by LCA under CR conditions. We found that each of these mutations significantly decreases the extent to which LCA can prolong both the mean and maximum CLS (Figures 9A, 9E, 9I and 9J for *cat2Δ*; Figures 9B, 9F, 9I and 9J for *crc1Δ*; Figures 9C, 9G, 9I and 9J for *yat1Δ*; and Figures 9D, 9H, 9I and 9J for *yat2Δ*). We therefore concluded that the carnitine-dependent transport of acetyl-CoA from peroxisomes to mitochondria makes an essential contribution to the LCA-dependent delay of yeast chronological aging under CR conditions.

### The peroxisome-to-mitochondrion transport of acetyl-CoA in the forms of the glyoxylate cycle intermediates citrate and succinate is dispensable for the delay of yeast chronological aging by LCA

Our previous studies have revealed that, during PD and ST phases of culturing yeast under CR on 0.2% glucose, LCA decreases the concentrations of the Cit2, Ctp1 and Dic1 proteins [36, 50, 53]. Cit2 is known to catalyze a peroxisomal reaction of the glyoxylate cycle leading to the conversion of acetyl-CoA into citrate [82] (Supplementary Figure 2). Ctp1 has been implicated in the delivery of this peroxisomally produced citrate to mitochondria, whereas Dic1 has been shown to be a mitochondrial transporter for another intermediate of the glyoxylate cycle, succinate [83]; both these transport proteins enable the replenishment of TCA cycle intermediates in mitochondria [84] (Supplementary Figure 2). We sought to determine whether the transport of acetyl-CoA from peroxisomes to mitochondria in the forms of citrate and succinate plays a role in the LCA-dependent delay of yeast chronological aging under CR conditions. We therefore assessed how a single-gene-deletion mutation eliminating the Cit2, Ctp1 or Dic1 protein affects the extent to which LCA can increase yeast CLS under CR conditions. We found that none of these mutations has a significant effect on the efficiency with which LCA can extend CLS under CR conditions (Supplementary Figures 6A, 6D, 6G and 6H for *cit2Δ*; Supplementary Figures 6B, 6E, 6G and 6H for *ctp1Δ*; and Supplementary Figures 6C, 6F, 6G and 6H for *dic1Δ*). Hence, in yeast limited in calorie supply, the peroxisome-to-mitochondrion transport of acetyl-CoA in the forms of the glyoxylate cycle intermediates citrate and succinate does not make an essential contribution to the delay of yeast chronological aging by LCA.

### The Mpc1/Mpc3 mitochondrial pyruvate carrier is necessary for the delay of yeast chronological aging by LCA

As we have previously found, during PD and ST phases of culturing yeast under CR on 0.2% glucose, LCA exhibits the following effects [36, 50, 53]: 1) it rises the concentrations of Mpc1 and Mpc3, the two protein components of a mitochondrial pyruvate carrier involved in pyruvate transport to mitochondria during respiratory growth [85, 86]; and 2) it does not change the concentration of the Mpc2 protein component of the mitochondrial pyruvate carrier Mpc1/Mpc2v formed during fermentative growth [85, 86] (Supplementary Figure 2). Based on these findings, we hypothesized that the Mpc1/Mpc2 and/ or Mpc1/Mpc3 mitochondrial pyruvate carriers may contribute to the LCA-dependent delay of yeast chronological aging under CR conditions. To test this hypothesis, we examined the effect of a single-gene-deletion mutation eliminating the Mpc1, Mpc2 or Mpc3 protein on the efficiency with which LCA can increase yeast CLS under CR conditions. We found the following: 1) the *mpc1Δ* and *mpc3Δ* mutations decrease the efficiency of yeast CLS extension by LCA (Supplementary Figures 7A, 7D, 7G and 7H for *mpc1Δ*; and Supplementary Figures 7C, 7F, 7G and 7H for *mpc3Δ*); and 2) the *mpc2Δ* mutation does not affect such efficiency (Supplementary Figures 7B, 7E, 7G and 7H). These findings indicate that the Mpc1/Mpc3 mitochondrial pyruvate carrier (which is formed during respiratory growth) plays an essential role in the LCA-dependent delay of chronological aging in yeast limited in calorie supply. In contrast, the Mpc1/Mpc2 mitochondrial pyruvate carrier (which is formed during fermentative growth) is dispensable for the delay of yeast chronological aging by LCA under CR conditions.

### The ability of LCA to shift a balance between the processes of mitochondrial fusion and fission toward fusion is essential for the delay of yeast chronological aging by LCA

**Figure 10:**
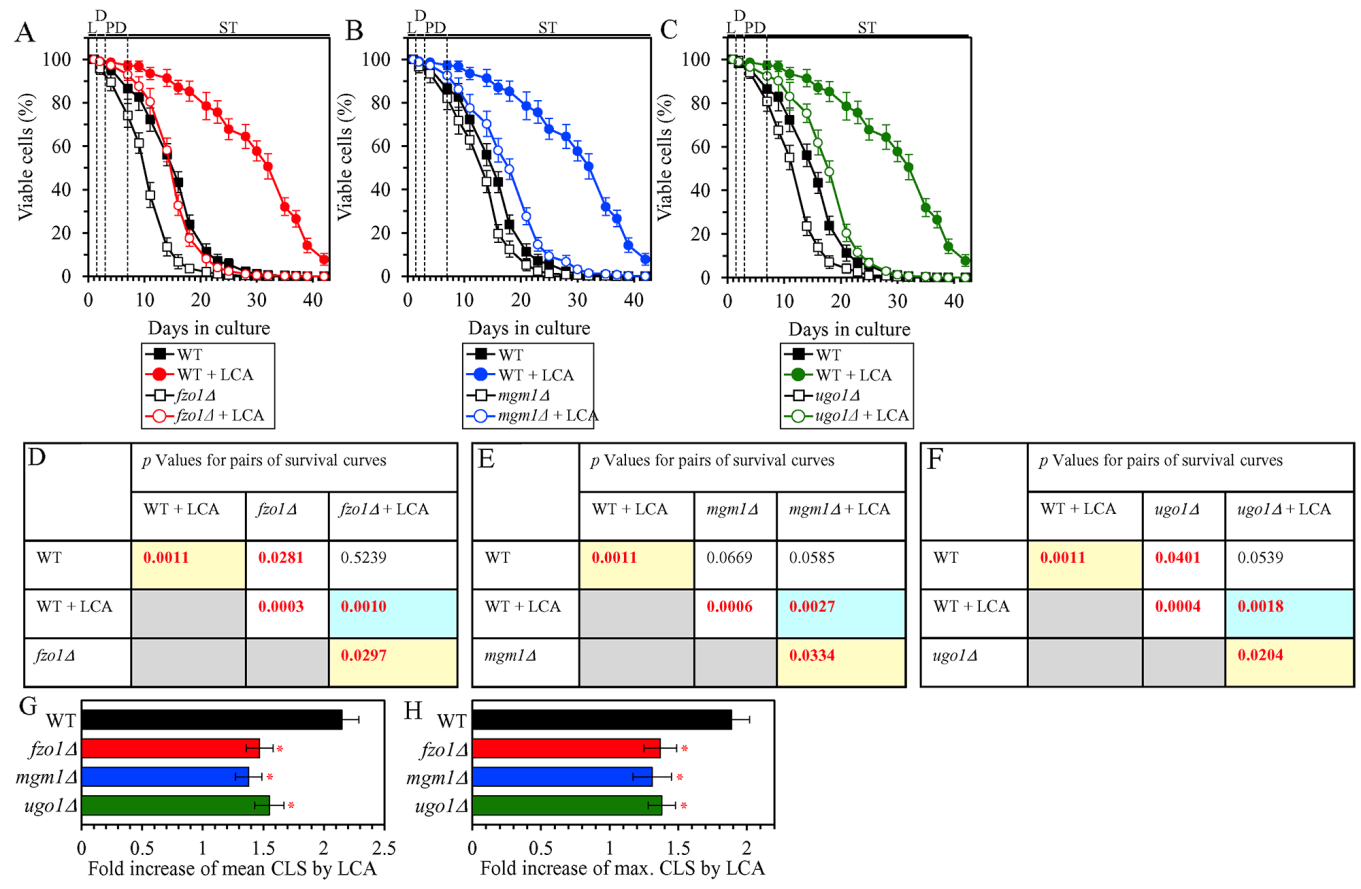
Under CR conditions in the presence of LCA, lack of the Fzo1, Mgm1 or Ugo1 protein component of the mitochondrial fusion machine decreases the efficiency of yeast CLS extension by LCA WT cells and mutant cells carrying a single-gene-deletion mutation eliminating either the Fzo1, Mgm1 or Ugo1 protein were cultured in the nutrient-rich YP medium initially containing 0.2% glucose with 50 μM LCA or without it. **(A, B, C)** Survival curves of the chronologically aging WT and *fzo1Δ* (A), WT and *mgm1Δ* (B) or WT and *ugo1Δ* (C) strains are shown. Data are presented as means ± SEM (n = 3). Data for the WT strain cultured with or without LCA are replicated in graphs A, B, C of this Figure. **(D, E, F)** *p* Values for different pairs of survival curves of the WT and *fzo1Δ* (D), WT and *mgm1Δ* (E) or WT and *ugo1Δ* (F) strains cultured with or without LCA. Survival curves shown in (A, B or C, respectively) were compared. Two survival curves were considered statistically different if the *p* value was less than 0.05. The *p* values for comparing pairs of survival curves using the logrank test were calculated as described in Materials and Methods. The *p* values displayed on a yellow color background indicate that LCA extends the CLS of the WT, *fzo1Δ* (D), *mgm1Δ* (E) and *ugo1Δ* (F) strains. The *p* values displayed on a blue color background in D, E and F indicate that LCA extends the CLS of the *fzo1Δ* (D), *mgm1Δ* (E) and *ugo1Δ* (F) strains to a lower extent than that of the WT strain. **(G, H)** Survival curves shown in (A, B, C) were used to calculate the fold of increase of the mean (G) and maximum (H) CLS by LCA for the WT, *fzo1Δ*, *mgm1Δ* and *ugo1Δ* strains. Data are presented as means ± SEM (n = 3; ^*^p < 0.05). Abbreviations: L, D, PD and ST, logarithmic, diauxic, post-diauxic and stationary growth phases (respectively).

**Figure 11:**
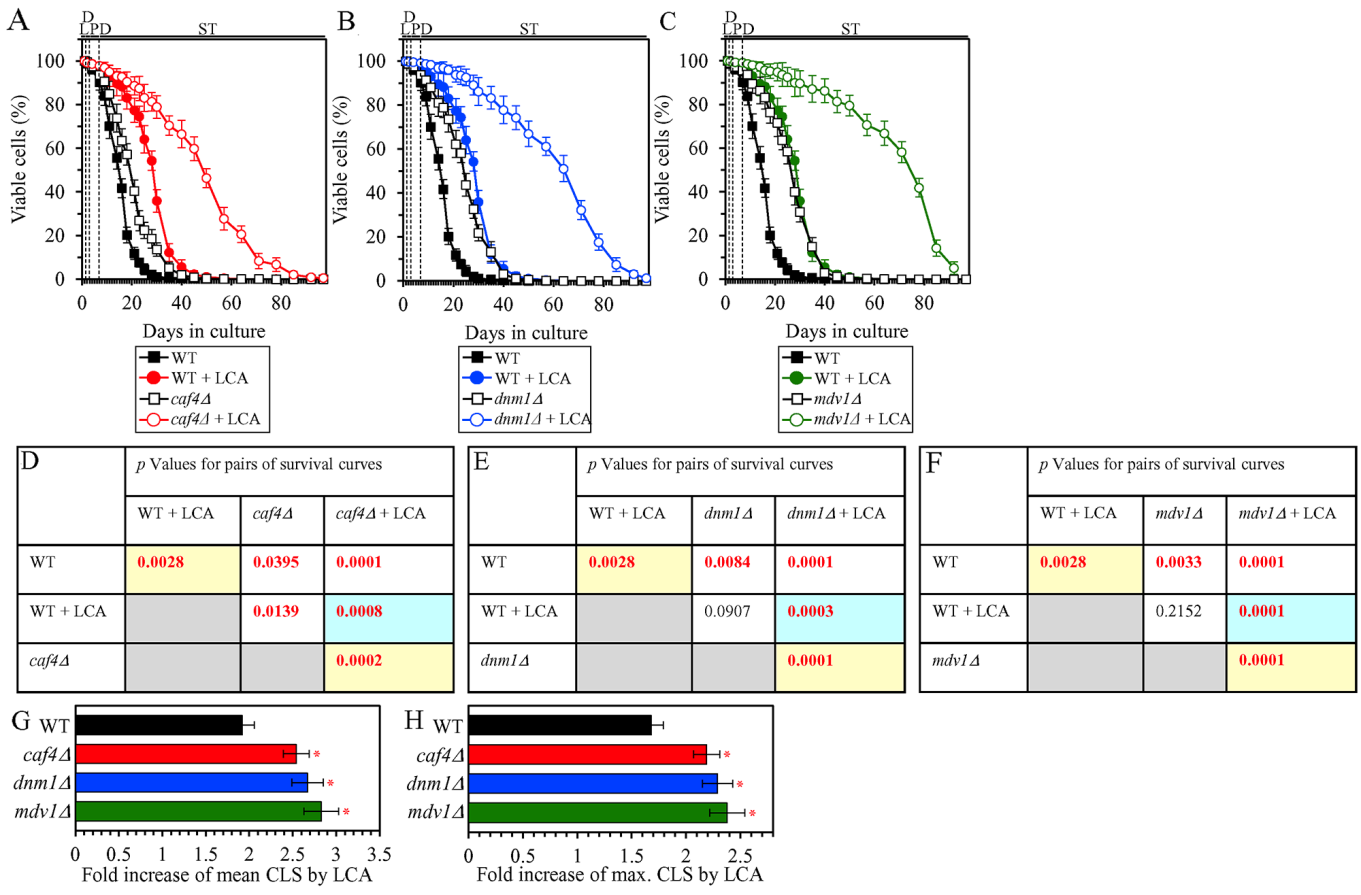
Under CR conditions in the presence of LCA, lack of the Caf4, Dnm1 or Mdv1 protein component of the mitochondrial fission machine increases the efficiency of yeast CLS extension by LCA WT cells and mutant cells carrying a single-gene-deletion mutation eliminating either the Caf4, Dnm1 or Mdv1 protein were cultured in the nutrient-rich YP medium initially containing 0.2% glucose with 50 μM LCA or without it. **(A, B, C)** Survival curves of the chronologically aging WT and *caf4Δ* (A), WT and *dnm1Δ* (B) or WT and *mdv1Δ* (C) strains are shown. Data are presented as means ± SEM (n = 3). Data for the WT strain cultured with or without LCA are replicated in graphs A, B, C of this Figure. **(D, E, F)** *p* Values for different pairs of survival curves of the WT and *caf4Δ* (D), WT and *dnm1Δ* (E) or WT and *mdv1Δ* (F) strains cultured with or without LCA. Survival curves shown in (A, B or C, respectively) were compared. Two survival curves were considered statistically different if the *p* value was less than 0.05. The *p* values for comparing pairs of survival curves using the logrank test were calculated as described in Materials and Methods. The *p* values displayed on a yellow color background indicate that LCA extends the CLS of the WT, *caf4Δ* (D), *dnm1Δ* (E) and *mdv1Δ* (F) strains. The *p* values displayed on a blue color background in D, E and F indicate that LCA extends the CLS of the *caf4Δ* (D), *dnm1Δ* (E) and *mdv1Δ* (F) strains to a higher extent than that of the WT strain. **(G, H)** Survival curves shown in (A, B, C) were used to calculate the fold of increase of the mean (G) and maximum (H) CLS by LCA for the WT, *caf4Δ*, *dnm1Δ* and *mdv1Δ* strains. Data are presented as means ± SEM (n = 3; ^*^p < 0.05). Abbreviations: L, D, PD and ST, logarithmic, diauxic, post-diauxic and stationary growth phases (respectively).

We have previously noticed that, during PD and ST phases of culturing yeast under CR on 0.2% glucose, LCA has the following effects on the morphology and protein composition of mitochondria: 1) it increases the percentage of cells exhibiting a tubular mitochondrial network [36]; 2) it decreases the percentage of cells displaying fragmented mitochondria [36]; 3) it causes a major enlargement of mitochondria [45]; 4) it significantly decreases mitochondrial number [45]; 5) it decreases the concentrations of the Caf4 and Mdv1 protein components of the mitochondrial fission machine [50]; and 6) it increases the concentrations of the Fzo1 and Ugo1 protein components of the mitochondrial fusion machine [53]. Because mitochondrial morphology is known to be defined by a balance between the processes of mitochondrial fusion and fission [87, 88], it is feasible that all these effects of LCA are due to its ability to shift this balance toward mitochondrial fusion. In support of this assumption, the mitochondrial membranes of LCA-treated yeast are enriched in PA [45, 53], a phospholipid known to increase mitochondrial size and to decrease mitochondrial number by causing the following two effects: 1) PA activates mitochondrial fusion [89–93]; in yeast treated with LCA, this effect of PA is due to its ability to stimulate the biogenesis of the Ugo1 protein component of the mitochondrial fusion machine [94]; and 2) PA inhibits mitochondrial fission because it decreases the efficiency with which the oligomerized dynamin-related protein component of the mitochondrial fission machine (Drp1 in mammals and Dnm1 in yeast) can constrict mitochondria [93, 95, 96]. We sought to determine whether the ability of LCA to shift a balance between the processes of mitochondrial fusion and fission toward fusion may play a role in the LCA-dependent delay of yeast chronological aging under CR conditions. We therefore examined how the extent to which LCA prolongs the CLS of yeast limited in calorie supply is influenced by the single-gene-deletion mutations eliminating protein components of the mitochondrial fusion or fission machine. We found the following: 1) the *fzo1Δ*, *mgm1Δ* and *ugo1Δ* mutations, each eliminating a protein component of the mitochondrial fusion machine, decrease the efficiency of yeast CLS extension by LCA (Figures 10A, 10D, 10G and 10H for *fzo1Δ*; Figures 10B, 10E, 10G and 10H for *mgm1Δ*; and Figures 10C, 10F, 10G and 10H for *ugo1*Δ); and 2) the *caf4Δ*, *dnm1Δ* and *mdv1Δ* mutations, each eliminating a protein component of the mitochondrial fission machine, increase the efficiency of yeast CLS extension by LCA (Figures 11A, 11D, 11G and 11H for *caf4Δ*; Figures 11B, 11E, 11G and 11H for *dnm1Δ*; and Figures 11C, 11F, 11G and 11H for *mdv1Δ*). We therefore concluded that the ability of LCA to shift a balance between the processes of mitochondrial fusion and fission toward fusion makes an essential contribution to the LCA-dependent delay of chronological aging in yeast limited in calorie supply.

### LCA delays the age-related onset and slows progression of mitochondria-controlled apoptosis and decreases cell susceptibility to this form of RCD in an age-dependent manner

**Figure 12:**
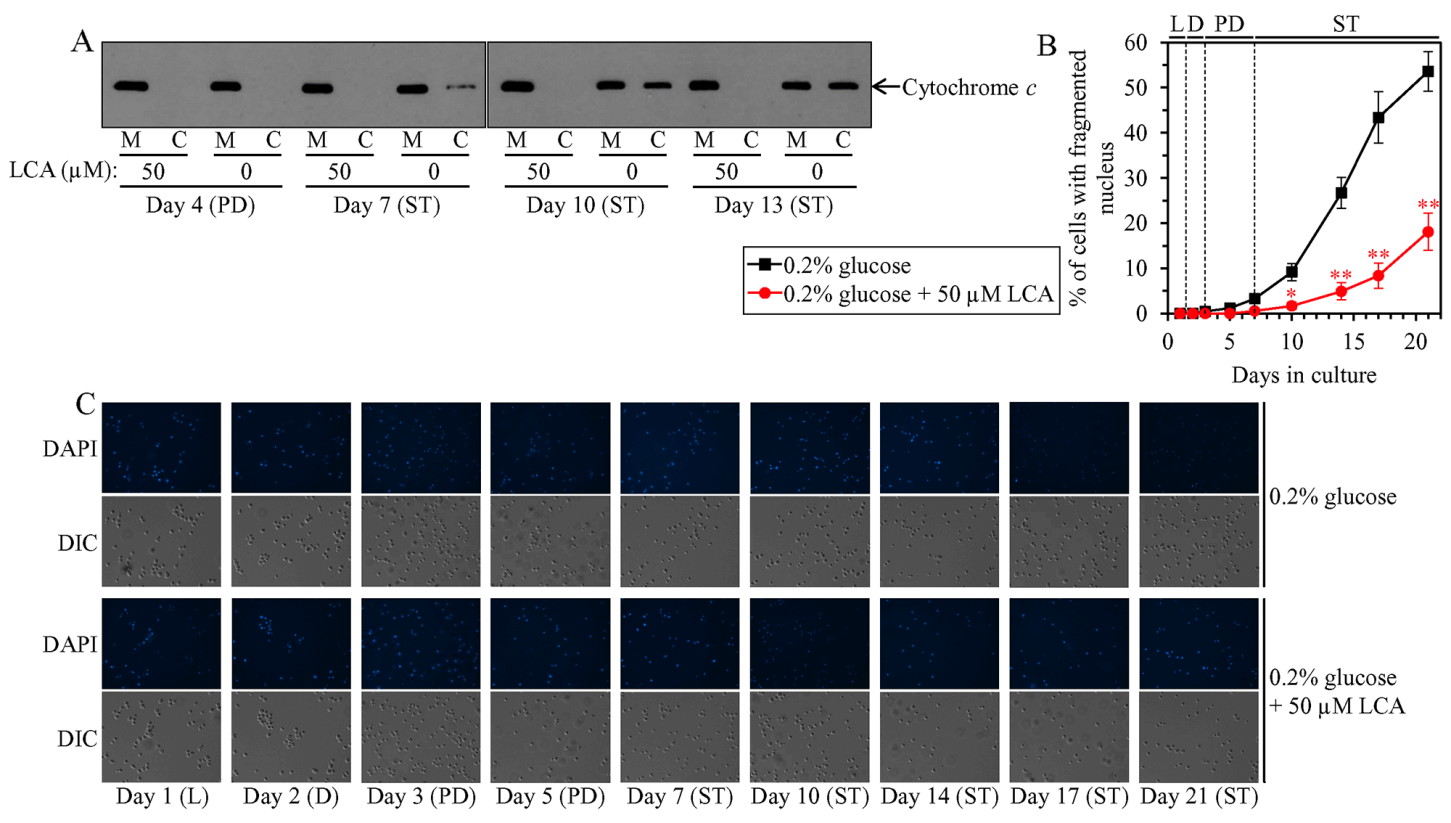
Under CR conditions, LCA postpones the age-related onset and decelerates progression of such late events in apoptotic RCD as cytochrome *c* efflux from mitochondria and nuclear fragmentation WT cells were cultured in the nutrient-rich YP medium initially containing 0.2% glucose with 50 μM LCA or without it. **(A)** Western blot analysis of cytochrome *c* in purified mitochondria (M) and in the cytosolic fraction (C) recovered from cells that were taken on different days of culturing. Equal portions of mitochondrial and cytosolic fractions were analyzed by immunoblotting to cytochrome *c*. **(B)** Percentage of cells exhibiting fragmented nucleus, a characteristic trait of apoptotic RCD. Images like the representative images shown in (C) were quantitated as described in Materials and Methods. Data are presented as means ± SEM (n = 3; ^*^p < 0.05; ^**^p < 0.01). **(C)** Cells recovered on different days of culturing were visualized using the DIC microscopy and stained with 4′,6-diamidino-2-phenylindole dihydrochloride (DAPI) to detect nuclei as described in Materials and Methods. Abbreviations: L, D, PD and ST, logarithmic, diauxic, post-diauxic and stationary growth phases (respectively).

**Figure 13:**
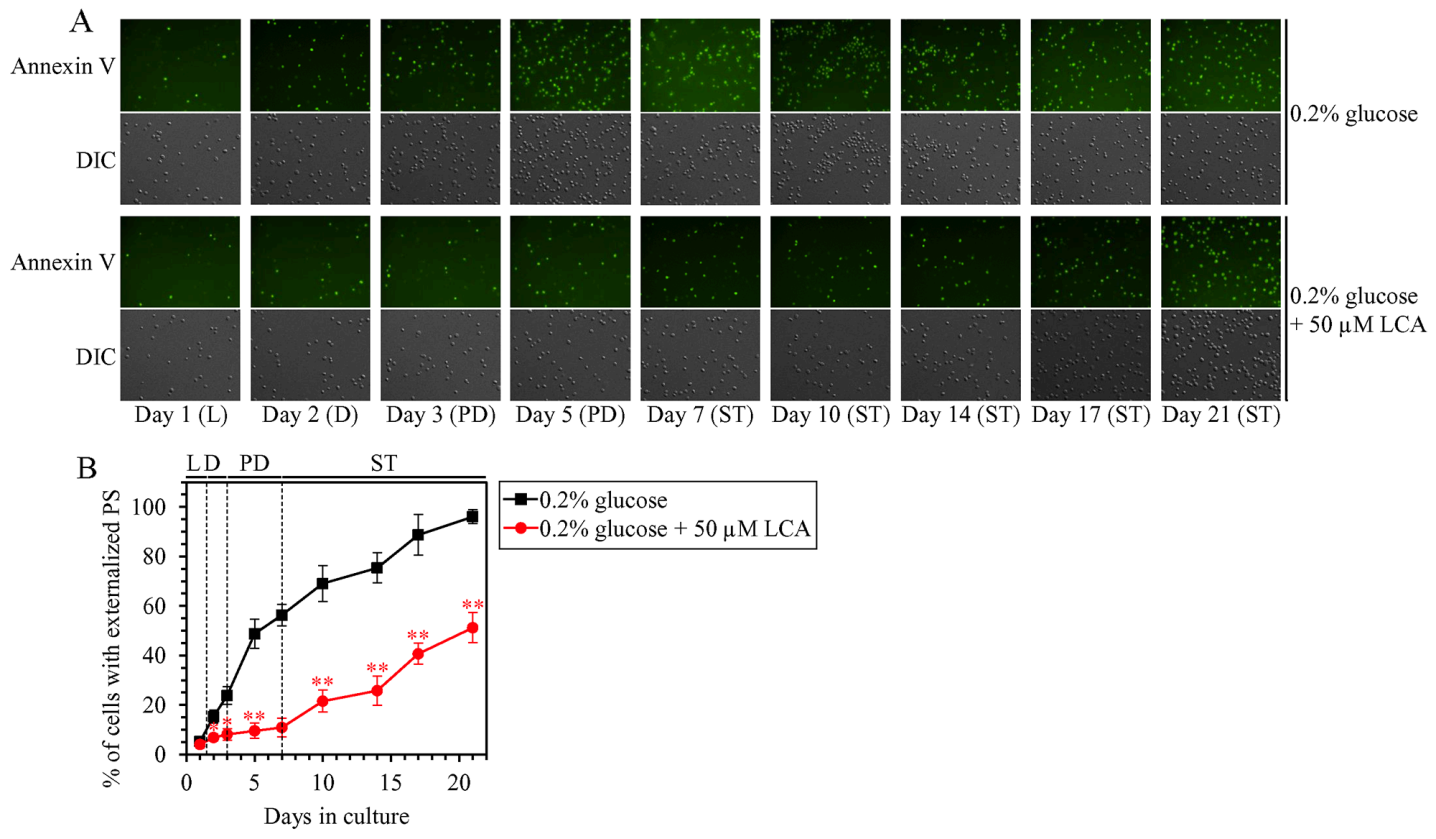
Under CR conditions, LCA postpones the age-related onset and slows progression of such early event in apoptotic RCD as phosphatidylserine (PS) translocation from the inner to the outer leaflet of the plasma membrane WT cells were cultured in the nutrient-rich YP medium initially containing 0.2% glucose with 50 μM LCA or without it. **(A)** Cells recovered on different days of culturing were visualized using the DIC microscopy and stained with Annexin V to detect the presence of PS in the outer leaflet of the plasma membrane as described in Materials and Methods. **(B)** Percentage of cells exhibiting externalized PS, a characteristic trait of apoptotic RCD consisting in the translocation of PS from the inner to the outer leaflet of the plasma membrane. Images like the representative images shown in (A) were quantitated as described in Materials and Methods. Data are presented as means ± SEM (n = 3; ^*^p < 0.05; ^**^p < 0.01). Abbreviations: L, D, PD and ST, logarithmic, diauxic, post-diauxic and stationary growth phases (respectively).

Mitochondrial network fragmentation is known as one of the hallmarks of a mitochondria-controlled form of age-related apoptotic RCD [73, 75, 97–107]. We have demonstrated that, during PD and ST phases of culturing yeast under CR on 0.2% glucose, LCA attenuates mitochondrial network fragmentation [36, 45, 50, 53] by shifting a balance between the processes of mitochondrial fusion and fission toward fusion (this study), and that this effect of LCA is essential for its ability to delay yeast chronological aging under CR conditions (this study). We therefore sought to determine whether in yeast limited in calorie supply LCA may affect age-related changes in some other hallmark events of this mitochondria-controlled form of apoptotic RCD. We found that the ability of LCA to delay mitochondrial network fragmentation during PD and ST phases coincides with its ability to postpone the age-related onset and decelerate progression of such characteristic event in apoptotic RCD as cytochrome *c* efflux from mitochondria during ST phase (Figure 12A); cytochrome *c* is known as a pro-apoptotic protein whose release from fragmented mitochondria into the cytosol of yeast cells is a late event in mitochondria-controlled apoptotic RCD [73, 75, 97–107]. We also found that LCA delays the age-related onset and slows progression of nuclear fragmentation during ST phase (Figure 12B and 12C); nuclear fragmentation in yeast is known as another characteristic late event of age-related apoptotic RCD [73, 75, 97–107]. Moreover, we noticed that LCA postpones the age-related onset and slows progression of phosphatidylserine (PS) translocation from the inner to the outer leaflet of the plasma membrane during PD and ST phases (Figure 13A and 13B); this so-called externalization of PS in yeast is known as an early event in age-related apoptotic RCD [108, 109].

Mitochondrial network fragmentation and the efflux of several pro-apoptotic proteins (including cytochrome *c*) from the intermediate space of fragmented mitochondria are known to be characteristic traits of mitochondria-controlled apoptotic RCD induced by a short-term exposure of yeast cells to exogenous hydrogen peroxide [73, 75, 97–107]. Our assessment of how LCA influences cell susceptibility to this mode of apoptotic RCD under CR conditions has revealed the following: 1) LCA significantly increases clonogenic survival of cells briefly (for 2 h) treated with hydrogen peroxide if these cells were recovered during PD or ST phase of culturing (Supplementary Figure 8); and 2) LCA does not alter clonogenic survival of cells subjected to such treatment if these cells were recovered during L or D phase of culturing (Supplementary Figure 8).

In sum, the above findings indicate that LCA delays yeast chronological aging in part because it attenuates mitochondrial network fragmentation, thereby delaying the onset of an age-related mode of mitochondria-controlled apoptotic RCD during PD and ST phases of culturing under CR conditions.

## DISCUSSION

**Figure 14:**
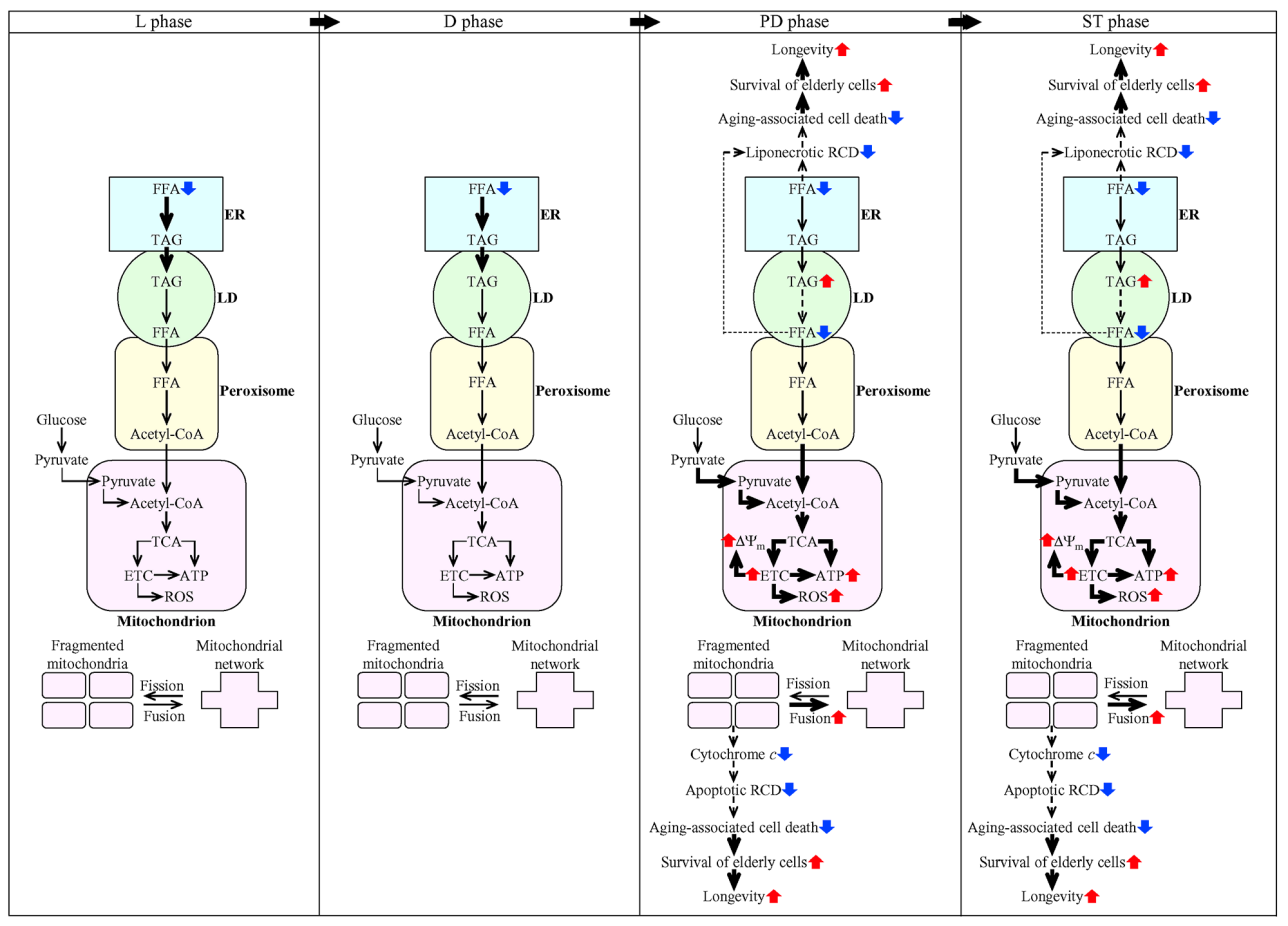
Mechanisms through which LCA delays chronological aging of yeast limited in calorie supply Inyeast cultured under CR conditions, LCA elicits an aging-delaying cellular pattern and preserves such pattern throughout the entire chronological lifespan, before an arrest of cell growth and division (i.e. during L, D and PD phases of culturing) and after such arrest (i.e. during ST phase of culturing). Under these conditions, LCA causes the stepwise development and maintenance of the aging-delaying cellular pattern because it alters the spatiotemporal dynamics of a cellular network integrating certain pathways of lipid and carbohydrate metabolism, some intercompartmental communications, specific aspects of mitochondrial morphology and functionality, and liponecrotic and apoptotic modes of RCD. Because LCA decreases the risk of aging-associated cell death to increase the chance of elderly cells to survive, it extends longevity of chronologically aging yeast. The thickness of black arrows is proportional to the rates of processes. Arrows next to the names of affected processes denote those of them that are intensified (red arrows) or weakened (blue arrows). Arrows next to the names of affected metabolites signify those of them whose concentrations are increased (red arrows) or decreased (blue arrows). Please see text for additional details. Abbreviations: Ac-CoA, acetyl-CoA; ER, endoplasmic reticulum; ETC, electron transport chain; FFA, free fatty acids; L, D, PD and ST, logarithmic, diauxic, post-diauxic and stationary growth phases (respectively); LD, lipid droplets; RCD, regulated cell death; ROS, reactive oxygen species; TAG, triacylglycerols; TCA, tricarboxylic acid cycle; ΔΨ_m_, mitochondrial electrochemical membrane potential.

This study and our previously published data [36, 45, 50, 53] suggest a hypothetical model for the mechanisms through which LCA delays chronological aging of yeast limited in calorie supply. This model is depicted in Figure 14. The key aspects of this model are as follows: 1) if LCA is added to culture medium at the time of cell inoculation, it elicits an aging-delaying cellular pattern and preserves such pattern throughout the entire chronological lifespan of the yeast culture, before an arrest of cell growth and division (i.e. during L, D and PD phases of culturing) and after such arrest (i.e. during ST phase of culturing); 2) LCA causes the stepwise development and maintenance of the aging-delaying cellular pattern in part because it alters various intercompartmental communications; these communications involve movements of certain intermediates of lipid and carbohydrate metabolism between the ER and LD, LD and peroxisomes, peroxisomes and mitochondria, the ER and mitochondria, and the cytosol and mitochondria; 3) LCA attenuates mitochondrial network fragmentation by shifting a balance between the processes of mitochondrial fusion and fission toward fusion; 4) the LCA-dependent changes in the communications between different cellular compartments and the LCA-driven weakening of mitochondrial network fragmentation decrease the risk of the cell commitment to liponecrotic and apoptotic RCD modes during PD and ST phases, thereby increasing the chance of cell survival during these phases of culturing; and 5) because LCA decreases the risk of aging-associated cell death to increase the chance of elderly cells to survive, it prolongs longevity of chronologically aging yeast (Figure 14).

Our findings indicate that LCA decreases the cellular concentration of FFA through the entire chronological lifespan of yeast limited in calorie supply. In our model, two mechanisms underlie this effect of LCA. One mechanism consists in the ability of LCA to regulate the anabolic branch of TAG metabolism by accelerating TAG synthesis from FFA within the ER and the ensuing TAG deposition within LD during L and D phases of culturing (Figure 14). The other mechanism involves an LCA-dependent regulation of the catabolic branch of TAG metabolism through the deceleration of TAG lipolysis into FFA within LD during PD and ST phases of culturing (Figure 14). By decreasing the cellular concentration of FFA through the entire chronological lifespan, LCA delays the age-related onset and decelerates the progression of FFA-induced liponecrotic RCD during PD and ST phases of culturing under CR conditions (Figure 14). This LCA-dependent decrease of the risk of aging-associated cell death increases the chance of elderly cells to survive and makes an essential contribution to the ability of LCA to extend longevity of chronologically aging yeast.

Our model further posits that the LCA-dependent stimulation of the peroxisome-to-mitochondrion transport of acetyl-CoA via the carnitine shuttle during PD and ST phases [50, 53] is indispensable for the delay of yeast chronological aging by LCA under CR conditions (Figure 14). Another essential contributor to this aging-delaying effect of LCA is the Mpc1/Mpc3 mitochondrial pyruvate carrier involved in the cytosol-to-mitochondrion transport of pyruvate during respiratory growth (Figure 14); akin to the carnitine-dependent transport of acetyl-CoA from peroxisomes to mitochondria, this mitochondrial pyruvate carrier is activated during PD and ST phases of culturing yeast under CR conditions [50, 53]. It is conceivable that these stimulating, aging-delaying effects of LCA on the peroxisome-to-mitochondrion transport of acetyl-CoA and the cytosol-to-mitochondrion transport of pyruvate may be involved in the following cause-effect relationship with other mitochondrial processes known to be elicited by LCA: 1) these effects may be caused by the accumulation of LCA in both mitochondrial membranes and by the subsequent remodeling of mitochondrial membrane lipidome during PD and ST phases of culturing under CR conditions [45, 47, 48, 52, 54]; and 2) these effects may cause the LCA-dependent changes in some vital aspects of mitochondrial functionality; these changes include a rise in mitochondrial respiration/electron transport chain, electrochemical membrane potential, reactive oxygen species concentration and ATP synthesis during PD and ST phases of culturing under CR conditions [45, 47, 48, 52, 54].

LCA is known to inhibit mitochondrial network fragmentation during PD and ST phases of culturing yeast under CR conditions [36, 45, 50, 53]. This study provides evidence that, during these phases of culturing, such effect of LCA on mitochondrial morphology is in the following cause-effect relationship with other cellular processes affected by LCA: 1) it is caused by the ability of LCA to shift a balance between the processes of mitochondrial fusion and fission toward fusion; 2) it causes a delay in the onset and a deceleration in the progression of an age-related mode of mitochondria-controlled apoptotic RCD (Figure 14). Like for the FFA-induced liponecrotic mode of RCD, the LCA-dependent decrease of the risk of aging-associated apoptotic mode of RCD increases the chance of elderly cells to survive and, thus, is an important contributing factor to longevity extension by LCA in chronologically aging yeast limited in calorie supply (Figure 14).

In sum, this study provides important new insights into the mechanisms by which LCA delays yeast chronological aging under CR conditions by altering the spatiotemporal dynamics of a cellular network that integrates certain pathways of lipid and carbohydrate metabolism, some intercompartmental communications, specific aspects of mitochondrial morphology and functionality, and liponecrotic and apoptotic modes of RCD. In the future, it would be interesting to investigate how LCA influences a novel mechanism of longevity extension by CR in chronologically aging yeast. This recently discovered mechanism has been shown to link cellular aging to cell cycle regulation, maintenance of a quiescent state, entry into a non-quiescent state and survival in the non-quiescent state [110].

## MATERIALS AND METHODS

### Yeast strains, media and growth conditions

The WT strain BY4742 (*MAT*α *his3Δ1 leu2Δ0 lys2Δ0 ura3Δ0*) and single-gene-deletion mutant strains in the BY4742 genetic background (all from Open Biosystems/Dharmacon, a part of GE Healthcare) were grown in YP medium (1% yeast extract, 2% peptone; both from Fisher Scientific; #BP1422-2 and #BP1420-2, respectively) initially containing 0.2% glucose (#D16-10; Fisher Scientific) with 50 μM LCA (#L6250; Sigma) or without it. Cells were cultured at 30°C with rotational shaking at 200 rpm in Erlenmeyer flasks at a “flask volume/medium volume” ratio of 5:1.

### Chronological life span assay

A sample of cells was taken from a culture at a certain time-point. A fraction of the sample was diluted to determine the total number of cells using a hemocytometer. Another fraction of the cell sample was diluted and serial dilutions of cells were plated in duplicate onto YP plates containing 2% glucose as carbon source. After 2 d of incubation at 30°C, the number of colony forming units (CFU) per plate was counted. The number of CFU was defined as the number of viable cells in a sample. For each culture, the percentage of viable cells was calculated as follows: (number of viable cells per ml/total number of cells per ml) × 100. The percentage of viable cells in mid-logarithmic phase was set at 100%. The life span curves were validated using a LIVE/DEAD yeast viability kit (Invitrogen) following the manufacturer’s instructions.

### Mass spectrometric identification and quantitation of cellular lipids

Extraction of cellular lipids and their mass spectrometric identification and quantitation were performed as previously described [111]. Briefly, a sample of cells was taken from a culture on a certain day of culturing. A fraction of the sample was diluted to determine the total number of cells using a hemocytometer (# 3200; Hausser Scientific). 5 × 10^7^ cells were harvested by centrifugation in a Centra CL2 clinical centrifuge for 5 min at 3,000 × g at room temperature. The cell pellet was washed once in ice-cold nanopure water and once in ice-cold 155 mM ammonium bicarbonate (pH 8.0), and the cells were harvested by centrifugation at 16,000 × g for 1 min at 4°C. The cell pellet was stored at -80°C until lipid extraction. For lipid extraction, the pelleted cells kept at -80°C were thawed on ice before being resuspended in 200 μl of ice-cold nanopure water. The re-suspended sample was transferred to a 15-ml high-strength glass screw top centrifuge tube with a Teflon lined cap (#0556912; Fisher Scientific). The volume of each sample was topped off to 1 ml with ice-cold nanopure water. To each tube the following was added:20 μL of the internal standard mix prepared in Chromasolv HPLC (>99.9%) chloroform (Sigma-Aldrich) as described [111], 800 μl of 425-600 μM acid-washed glass beads to break open the cells (#G8772; Sigma-Aldrich) and 3 ml of a Chromasolv HPLC (>99.9%) chloroform-methanol mixture (both from Sigma-Aldrich) at a 17:1 ratio. The samples were then vortexed vigorously for 2 h at 4°C and subjected to centrifugation in a Centra CL2 clinical centrifuge at 3,000 × g for 5 min at room temperature. The lower organic phase was then transferred to another 15-ml high-strength glass screw top centrifuge tube using a glass Pasteur pipette with careful attention not to disrupt the glass beads or upper aqueous phase. 1.5 ml of chloroform-methanol (2:1) solution was added to the remaining upper aqueous phase. The samples were again vortexed vigorously at 4°C for 2 h. The initial separated organic band was kept at 4°C for the duration of the second vortexing. At the end of 2-h vortexing, the samples were again centrifuged for 5 min at 3,000 × g at room temperature; the lower organic phase was then separated and added to the corresponding initial organic phase with a glass Pasteur pipette. With both lower organic phases combined, the solvent was evaporated off by nitrogen gas flow. Once all solvent was evaporated, the remaining lipid film was dissolved in 100 μl of chloroform-methanol (1:2) and immediately transferred into 2-ml glass vials with Teflon screw tops to avoid evaporation until samples were analyzed by mass spectrometry. Samples were then stored at -80°C and ran on the LTQ Orbitrap Mass Spectrometer within one week of the extraction. Samples were diluted (1:1) with chloroform/methanol (1:2) mixture supplemented with 0.1% ammonium hydroxide. Lipids were resolved by direct injection using a Thermo Orbitrap Velos mass spectrometer equipped with a HESI-II ion source (Thermo Scientific, Waltham, MA, USA) at a flow rate of 5 μl/min. The optimized tune setting and instrument methods for mass spectrometric analysis of lipids were previously described [111]. Mass spectra were converted to open format mzXML using the ProteoWizard MSConvert software (http://proteowizard.sourceforge.net/), the file format used by the Lipid Identification Software LipidXplorer (https://wiki.mpi-cbg.de/lipidx/Main_Page) for the automated detection and quantitation of lipid species. Data were normalized by taking the ratio of signal intensity of precursor ions to that of their respective lipid class-specific internal standard (spiked standard), multiplied by the concentration of that standard to give a molar quantity.

### Fluorescence microscopy

BODIPY 493/503 (#D3922, Thermo Fisher Scientific) staining for monitoring neutral lipids deposited in lipid droplets [73], propidium iodide (PI; #P4170, Sigma) staining for visualizing the extent of plasma membrane permeability for small molecules [112], 4′,6-diamidino-2-phenylindole dihydrochloride (DAPI; #D9542; Sigma) staining for visualizing nuclei [112] and Annexin V (#A13201; Thermo Fisher Scientific) staining for visualizing externalized phosphatidylserine [112] were performed according to established procedures. Live imaging was performed on a Leica DM6000B epifluorescence microscope equipped with a high-resolution Hamamatsu Orca ER CCD camera using oil immersion and a 100× objective. Images were acquired with 20-ms exposures using PerkinElmer Volocity software. Image files were exported as TIFFs then opened in ImageJ, where the percentage of BODIPY 493/503-, PI- and Annexin V-positive cells or cells with fragmented nucleus was calculated.

### Cell viability assay for monitoring the susceptibility of yeast to a mode of cell death induced by palmitoleic acid (POA)

A sample of cells was taken from a culture on a certain day of culturing. A fraction of the sample was diluted to determine the total number of cells using a hemocytometer. 8 × 10^7^ cells were harvested by centrifugation for 1 min at 21,000 × g at room temperature and resuspended in 8 ml of YP medium containing 0.2% glucose as carbon source. Each cell suspension was divided into 8 equal aliquots. Three pairs of aliquots were supplemented with POA (#P9417; Sigma) from a 50-mM stock solution (in 10% chloroform, 45% hexane and 45% ethanol; #650498, #248878 and #34852, respectively; all from Sigma). The final concentration of POA was 0.05 mM, 0.1 mM or 0.15 mM for each pair of aliquots; in all these aliquots, the final concentrations of chloroform, hexane and ethanol were 0.03%, 0.135% and 0.135%, respectively. One pair of aliquots was supplemented only with chloroform, hexane and ethanol added to the final concentrations of 0.03%, 0.135% and 0.135%, respectively. All aliquots were then incubated for 2 h at 30°C on a Labquake rotator (#400110; Thermolyne/Barnstead International) set for 360^o^ rotation. Serial dilutions of cells were plated in duplicate onto plates containing YP medium with 2% glucose as carbon source. After 2 d of incubation at 30°C, the number of colony forming units (CFU) per plate was counted. The number of CFU was defined as the number of viable cells in a sample. For each aliquot of cells exposed to POA, the % of viable cells was calculated as follows: (number of viable cells per ml in the aliquot exposed to POA/number of viable cells per ml in the control aliquot that was not exposed to POA) × 100.

### Cell viability assay for monitoring the susceptibility of yeast to a mode of cell death induced by hydrogen peroxide

A sample of cells was taken from a culture on a certain day of culturing. A fraction of the sample was diluted to determine the total number of cells using a hemocytometer. 8 × 10^7^ cells were harvested by centrifugation for 1 min at 21,000 × g at room temperature and resuspended in 8 ml of YP medium containing 0.2% glucose as carbon source. Each cell suspension was divided into 8 equal aliquots. Three pairs of aliquots were supplemented with hydrogen peroxide (#H325-500; Fisher Scientific) to the final concentration of 0.5 mM, 1.5 mM or 2.5 mM for each pair. One pair of aliquots remained untreated. All aliquots were then incubated for 2 h at 30°C on a Labquake rotator (#400110; Thermolyne/Barnstead International) set for 360^o^ rotation. Serial dilutions of cells were plated in duplicate onto plates containing YP medium with 2% glucose as carbon source. After 2 d of incubation at 30°C, the number of CFU per plate was counted. The number of CFU was defined as the number of viable cells in a sample. For each aliquot of cells exposed to hydrogen peroxide, the % of viable cells was calculated as follows: (number of viable cells per ml in the aliquot exposed to hydrogen peroxide/number of viable cells per ml in the control aliquot that was not exposed to hydrogen peroxide) × 100.

### Statistical analysis

Statistical analysis was performed using Microsoft Excel’s (2010) Analysis ToolPak - VBA. All data on cell survival are presented as mean ± SEM. The *p* values for comparing the means of two groups using an unpaired two-tailed *t* test were calculated with the help of the GraphPad Prism 7 statistics software. The logrank test for comparing each pair of survival curves was performed with GraphPad Prism 7. Two survival curves were considered statistically different if the *p* value was less than 0.05. The Pearson correlation coefficient (r) and the R-squared values were computed using Microsoft Excel’s (2010) Analysis ToolPak - VBA. The Pearson’s correlation coefficient (r) values ranging from 0.9 to 1.0 (-0.9 to -1.0) were considered a very high positive (negative) correlation between the two variables, whereas the Pearson’s correlation coefficient (r) values ranging from 0.7 to 0.9 (-0.7 to -0.9) were considered a high positive (negative) correlation between the two variables [74].

### Miscellaneous procedures

Subcellular fractionation of yeast and purification of mitochondria [113], and SDS-PAGE and immunoblotting [114] were performed as previously described.

## SUPPLEMENTARY MATERIALS FIGURES



## Abbreviations

CL: cardiolipin
CLS: chronological lifespan
CR: caloric restriction
DHAP: dihydroxyacetone phosphate
DIC: differential interference contrast
ER: endoplasmic reticulum
FA-CoA: fatty acyl-CoA ester
FFA: free fatty acids
Gro: glycerol
Gro-3-P: glycerol-3-phosphate
IMM: inner mitochondrial membrane
LCA: lithocholic acid
LD: lipid droplets
L, D, PD and ST: logarithmic, diauxic, post-diauxic and stationary growth phases (respectively)
LPA: lysophosphatidic acid
MAG: monoacylglycerol
MLCL: monolysocardiolipin
mtDNA: mitochondrial DNA
nDNA: nuclear DNA
OMM: outer mitochondrial membrane
PA: phosphatidic acid
PC: phosphatidylcholine
PDH: pyruvate dehydrogenase
PE: phosphatidylethanolamine
PG: phosphatidylglycerol
PI: phosphatidylinositol
POA: palmitoleic acid
PS: phosphatidylserine
RCD: regulated cell death
TAG: triacylglycerols
WT: wild-type
