# Supplementary material for: Mechanisms through which lithocholic acid delays yeast chronological aging under caloric restriction conditions
Source: Oncotarget. 2018 Oct 9;9(79):34945–71. doi: 10.18632/oncotarget.26188 (PMC6201858; doi:10.18632/oncotarget.26188)
Supplement: Supplementary file 1 [file oncotarget-09-34945-s001.pdf]

Mechanisms through which lithocholic acid delays yeast chronological aging under caloric restriction conditions

SUPPLEMENTARY MATERIALS

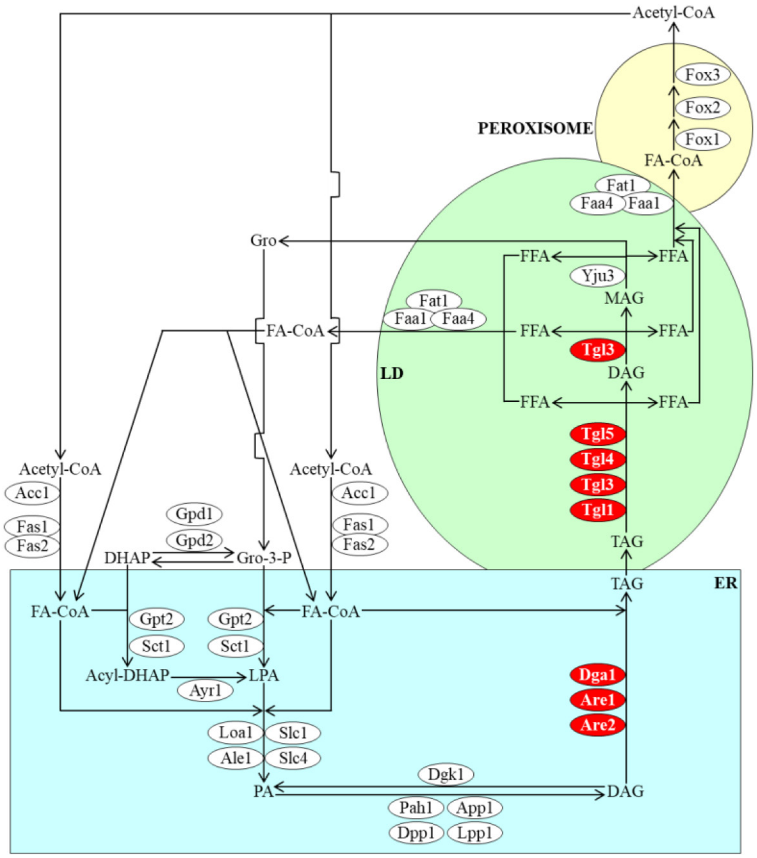

Supplementary Figure 1: The anabolic branch of TAG metabolism occurs in the endoplasmic reticulum (ER), whereas the catabolic branch of TAG metabolism is confined to lipid droplets (LD) and peroxisomes. TAG are ″neutral″ (uncharged) lipids initially synthesized in the ER from free fatty acids (FFA). FFA can also be formed as the products of TAG lipolysis in LD. Moreover, FFA can undergo β-oxidation in peroxisomes. Proteins displayed in red color: proteins that are eliminated by the single-gene-deletion mutations whose effects on yeast chronological lifespan, lipid concentrations, percent of cells exhibiting propidium iodide positive staining characteristic of necrotic cell death and cell viability following a short-term treatment with palmitoleic acid were studied. See text for more details. Abbreviations: Acc1, acetyl-CoA carboxylase 1; Ale1, acyltransferase for lysophosphatidylethanolamine 1; App1, actin patch protein 1; Are1/2, acyl-coenzyme A: cholesterol acyl transferase-related enzymes 1 and 2; Dga1, diacylglycerol acyltransferase 1; Dgk1, diacylglycerol kinase 1; Dpp1, diacylglycerol pyrophosphate phosphatase 1; DHAP, dihydroxyacetone phosphate; Faa1/4, fatty acid activation 1 and 2; FA-CoA, fatty acyl-CoA ester; Fas1/2, fatty acid synthetases 1 and 2; Fat1, fatty acid transporter 1; Fox1/2/3, fatty acid oxidation 1, 2 and 3; Gpd1/2, glycerol-3-phosphate dehydrogenases 1 and 2; Gpt2, glycerol-3-phosphate acyltransferase; Gro, glycerol; Gro-3-P, glycerol-3-phosphate; Loa1, lysophosphatidic acid: oleoyl-CoA acyltransferase 1; Lpp1, lipid phosphate phosphatase 1; LPA, lysophosphatidic acid; MAG, monoacylglycerol; PA, phosphatidic acid; Pah1, phosphatidic acid phosphohydrolase 1; Sct1, suppressor of choline-transport mutants 1; Slc1/4, sphingolipid compensation 1 and 4; Tgl1/3/4/5, triglyceride lipases 1, 3, 4 and 5.

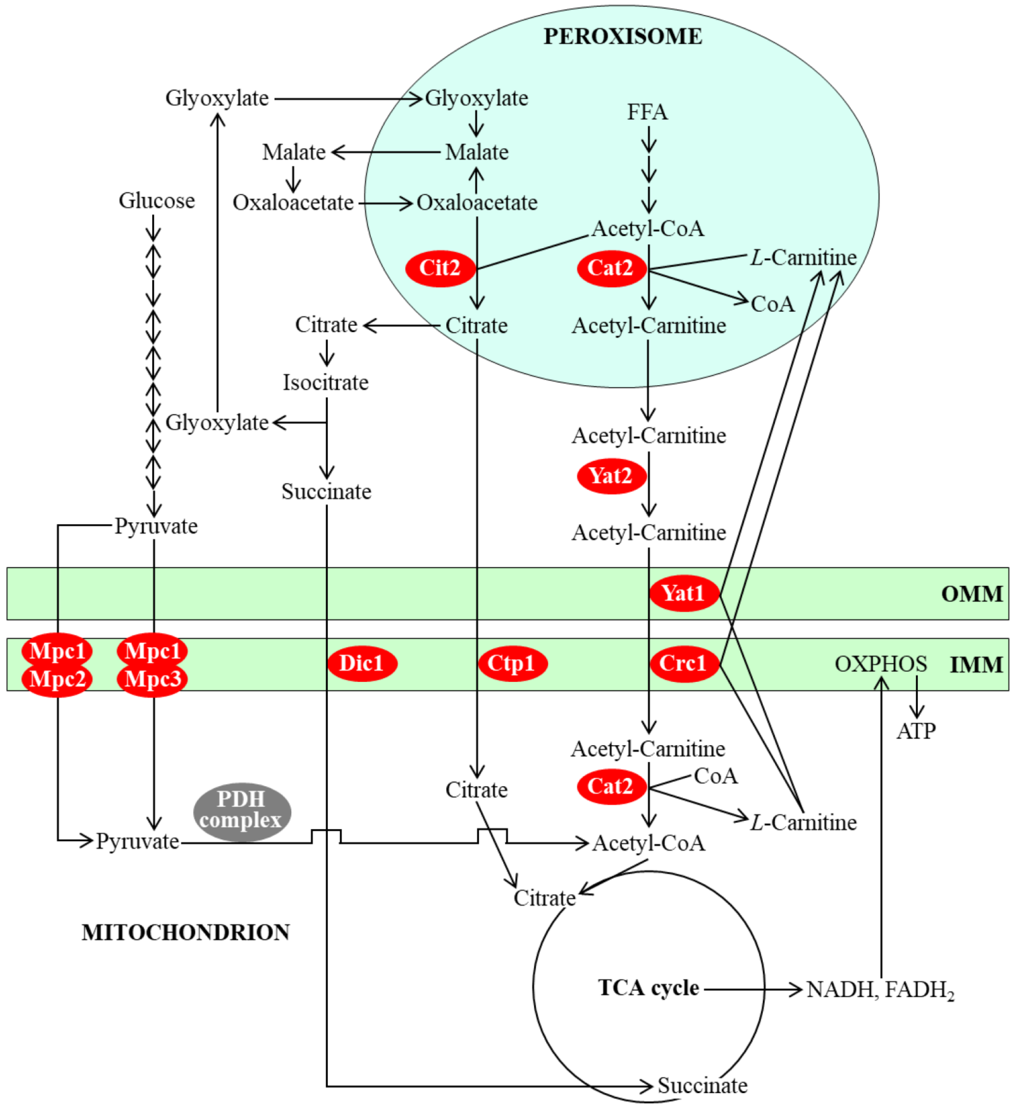

Supplementary Figure 2: As the final product of the β-oxidation of free fatty acids (FFA) in peroxisomes, acetyl-CoA: 1) is transported to mitochondria via the carnitine shuttle; 2) is oxidized in mitochondria through the TCA cycle, thus providing reducing equivalents NADH and $FADH_2$ for ATP synthesis via oxidative phosphorylation (OXPHOS); and 3) is transported to mitochondria in the forms of the glyoxylate cycle intermediates citrate and succinate to replenish the mitochondrial pool of TCA cycle intermediates. As the final product of glycolysis, pyruvate: 1) is transported to mitochondria via mitochondrial pyruvate carrier; and 2) is converted to acetyl-CoA, which is oxidized in mitochondria through the TCA cycle. Proteins displayed in red color: proteins that are eliminated by the single-gene-deletion mutations whose effects on yeast chronological lifespan were studied. See text for more details. Abbreviations: Cat2, carnitine acetyl-CoA transferase; Cit2, citrate synthase; Crc1, carnitine transporter; Ctp1, citrate transporter; Dic1, dicarboxylate carrier; Mpc1, Mpc2 and Mpc3, mitochondrial pyruvate carrier1 1, 2 and 3; PDH, pyruvate dehydrogenase; Yat1, mitochondrial carnitine acetyltransferase; Yat2, cytosolic carnitine acetyltransferase. IMM and OMM, inner and outer mitochondrial membranes.

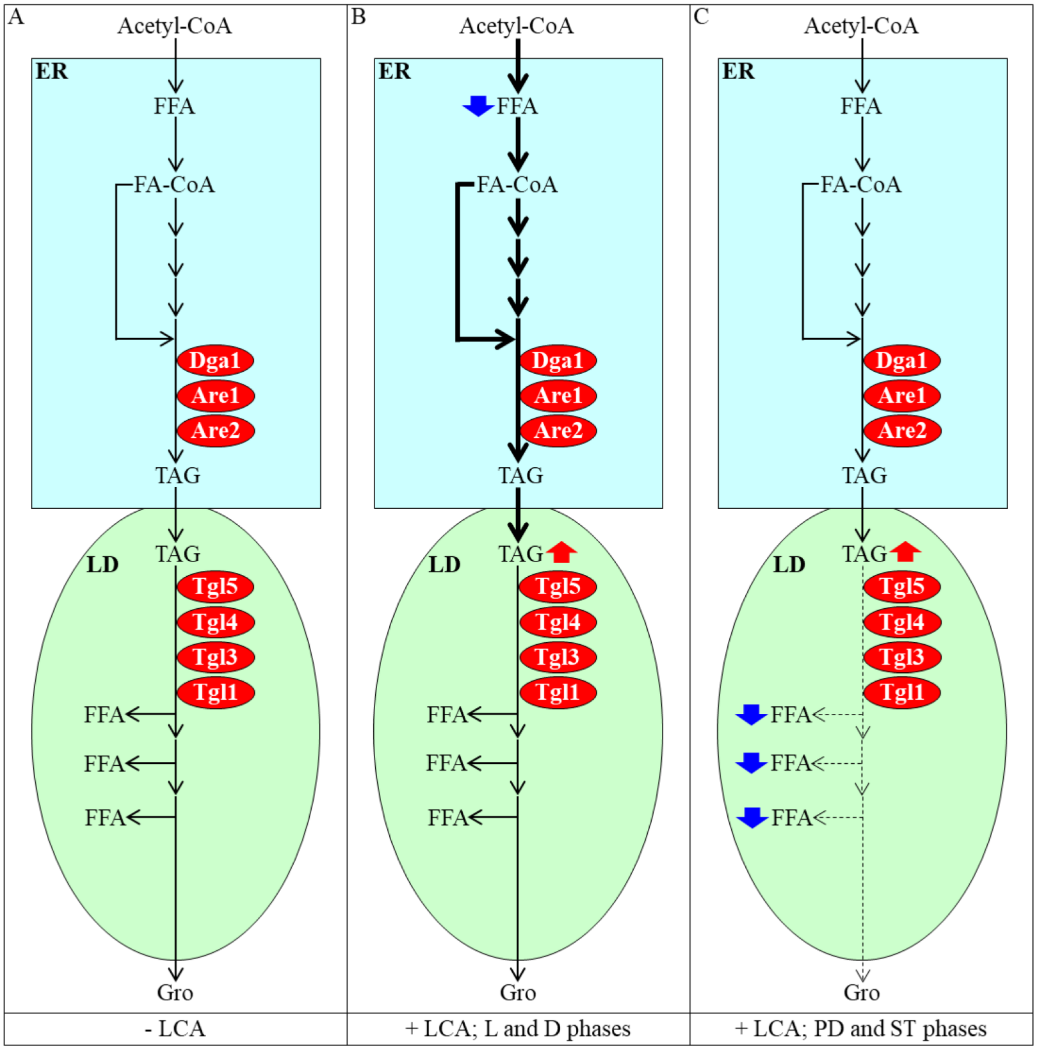

Supplementary Figure 3: Mechanism through which LCA regulates the anabolic branch of triacylglycerol (TAG) metabolism in the endoplasmic reticulum (ER) and the catabolic branch of TAG metabolism in lipid droplets (LD). (A) Relative rates of reactions and relative concentrations of metabolites in the anabolic branch of TAG metabolism in the ER and the catabolic branch of TAG metabolism in LD in yeast cells cultured under CR conditions without LCA. **(B)** During logarithmic (L) and diauxic (D) phases of culturing under CR conditions with LCA, this bile acid accelerates TAG synthesis from free fatty acids (FFA) within the ER and the subsequent TAG deposition within LD, thereby decreasing the concentration of FFA during these phases. **(C)** During post-diauxic (PD) and stationary (ST) phases of culturing under CR conditions with LCA, this bile acid decelerates TAG lipolysis into FFA within LD, thus decreasing the concentration of FFA during PD and ST phases. The thickness of black arrows is proportional to the rates of the corresponding metabolic reactions. Arrows next to the names of lipid classes denote those of them whose concentrations are increased (red arrows) or decreased (blue arrows) in yeast cultured under CR conditions with LCA. Abbreviations: Are1/2, acyl-coenzyme A: cholesterol acyl transferase-related enzymes 1 and 2; Dga1, diacylglycerol acyltransferase 1; FA-CoA, fatty acyl-CoA ester; Gro, glycerol; MAG, monoacylglycerol; Tgl1/3/4/5, triglyceride lipases 1, 3, 4 and 5.

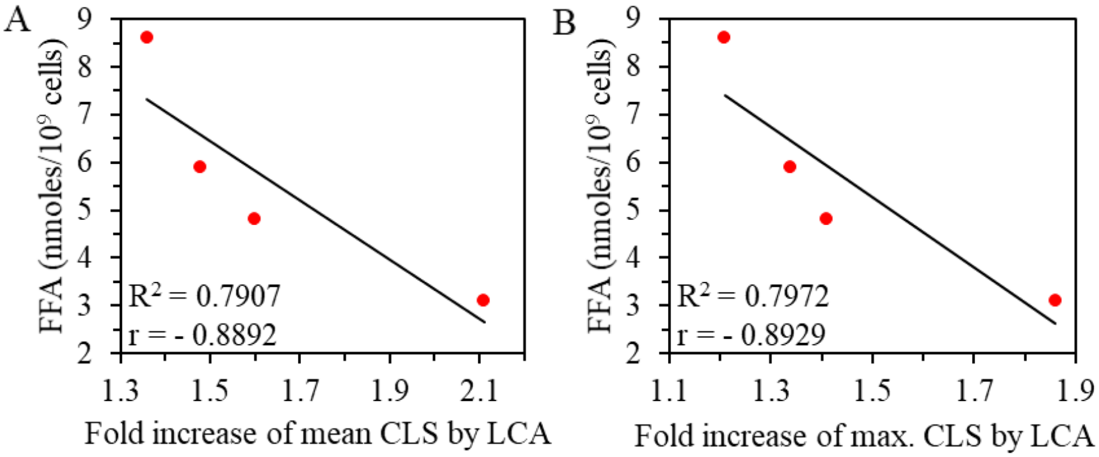

C

Genetic background	Fold increase of mean CLS by LCA	Fold increase of max. CLS by LCA	FFA concentration (nmoles/10^9 cells)
WT	2.11 ± 0.14	1.86 ± 0.09	3.1 ± 0.5
are2Δ	1.6 ± 0.09	1.41 ± 0.11	4.8 ± 0.3
dga1Δ	1.48 ± 0.07	1.34 ± 0.07	5.9 ± 0.6
are1Δ	1.36 ± 0.11	1.21 ± 0.12	8.6 ± 0.9

Supplementary Figure 4: Under CR conditions in the presence of LCA, lack of any of the three enzymes involved in the synthesis of TAG from FFA decreases the extent to which LCA can extend yeast CLS proportionally to the cellular concentration of FFA. WT cells and mutant cells carrying a single-gene-deletion mutation eliminating either Dga1, Are1 or Are2 were cultured in the nutrient-rich YP medium initially containing 0.2% glucose with 50 µM LCA. Survival curves shown in Figures 3A, 3F and 3K were used to calculate the folds of increase of the mean and maximum CLS by LCA for the WT and *dga1Δ*, *are1Δ* or *are2Δ* strains. **(A, B)** Plots comparing the folds increase of mean (A) or maximum (B) CLS and the maximum intracellular concentration of FFA (which was observed in WT and mutant cells recovered on day 2 of culturing with LCA). Different points show the data for WT, *dga1Δ*, *are1Δ* or *are2Δ* cells. Linear trendlines and the R-squared values are displayed; these values demonstrate a good fit of the line to the data. The Pearson's correlation coefficient (r) values are also shown; because the r value ranging from -0.7 to -0.9 is considered a high negative correlation between the two variables, the fold increase of the mean (A) or maximum (B) CLS has a high negative correlation with the intracellular concentration of FFA. **(C)** The experimental data used to create plots shown in (A and B). Genetic backgrounds of strains, the folds of increase of the mean and maximum CLS by LCA, and the maximum concentration of FFA (which was observed in WT and mutant cells recovered on day 2 of culturing with LCA) are shown. Data are presented as means ± SEM (n = 3). Abbreviation: FFA, free fatty acids.

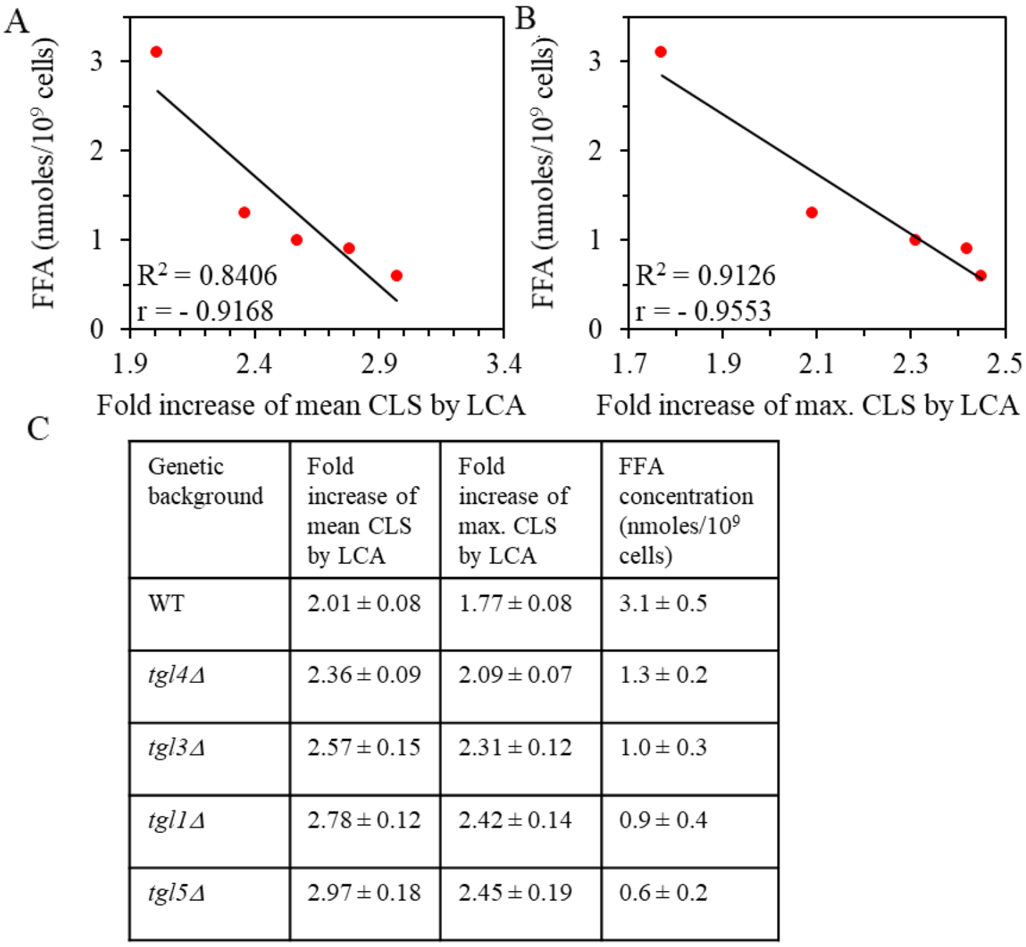

C

Genetic background	Fold increase of mean CLS by LCA	Fold increase of max. CLS by LCA	FFA concentration (nmoles/10^9 cells)
WT	2.01 ± 0.08	1.77 ± 0.08	3.1 ± 0.5
tgl4Δ	2.36 ± 0.09	2.09 ± 0.07	1.3 ± 0.2
tgl3Δ	2.57 ± 0.15	2.31 ± 0.12	1.0 ± 0.3
tgl1Δ	2.78 ± 0.12	2.42 ± 0.14	0.9 ± 0.4
tgl5Δ	2.97 ± 0.18	2.45 ± 0.19	0.6 ± 0.2

Supplementary Figure 5: Under CR conditions in the presence of LCA, lack of any of the four enzymes involved in the TAG lipolysis that yields FFA increases the extent to which LCA can extend yeast CLS proportionally to the cellular concentration of FFA. WT cells and mutant cells carrying a single-gene-deletion mutation eliminating either Tgl1, Tgl3, Tgl4 or Tgl5 were cultured in the nutrient-rich YP medium initially containing 0.2% glucose with 50 µM LCA. Survival curves shown in Figures 4A, 4F, 5A and 5F were used to calculate the folds of increase of the mean and maximum CLS by LCA for the WT, *tgl1Δ*, *tgl3Δ*, *tgl4Δ* and *tgl5Δ* strains. **(A, B)** Plots comparing the folds increase of mean (A) or maximum (B) CLS and the maximum intracellular concentration of FFA (which was observed in WT and mutant cells recovered on day 2 of culturing with LCA). Different points show the data for WT, *tgl1Δ*, *tgl3Δ*, *tgl4Δ* or *tgl5Δ* cells. Linear trendlines and the R-squared values are displayed; these values demonstrate a good fit of the line to the data. The Pearson's correlation coefficient (r) values are also shown; because the r value ranging from -0.9 to -1.0 is considered a very high negative correlation between the two variables, the fold increase of the mean (A) or maximum (B) CLS has a high negative correlation with the intracellular concentration of FFA. **(C)** The experimental data used to create plots shown in (A and B). Genetic backgrounds of strains, the folds of increase of the mean and maximum CLS by LCA, and the maximum concentration of FFA (which was observed in WT and mutant cells recovered on day 2 of culturing with LCA) are shown. Data are presented as means ± SEM (n = 3). Abbreviation: FFA, free fatty acids.

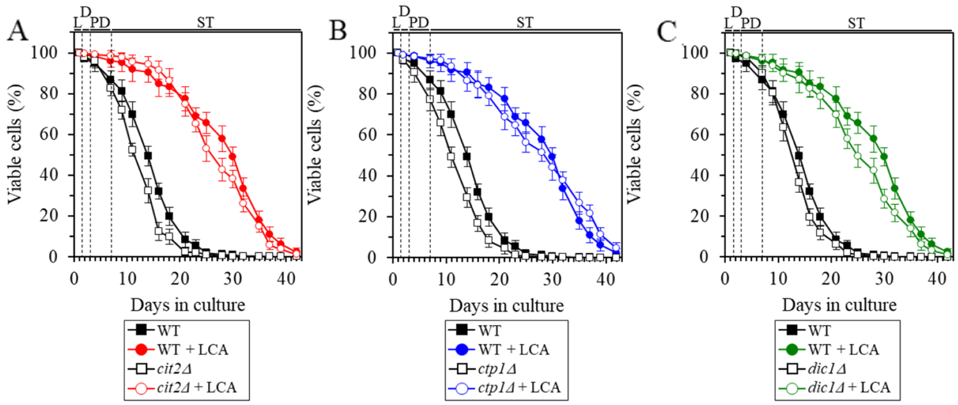

D

	p Values for pairs of survival curves		
	WT + LCA	*cit2Δ*	*cit2Δ* + LCA
WT	**0.0012**	0.0698	**0.0021**
WT + LCA		**0.0006**	0.0603
cit2Δ			**0.0005**

E

	p Values for pairs of survival curves		
	WT + LCA	*ctp1Δ*	*ctp1Δ* + LCA
WT	**0.0012**	0.0712	**0.0013**
WT + LCA		**0.0005**	0.0726
ctp1Δ			**0.0004**

F

	p Values for pairs of survival curves		
	WT + LCA	*dic1Δ*	*dic1Δ* + LCA
WT	**0.0012**	0.1208	**0.0032**
WT + LCA		**0.0007**	0.0576
dic1Δ			**0.0015**

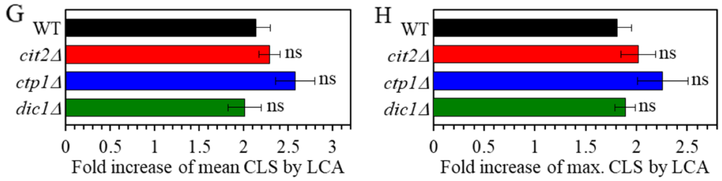

Supplementary Figure 6: Under CR conditions in the presence of LCA, lack of any of the three proteins required for the peroxisome-to-mitochondrion transport of acetyl-CoA in the forms of the glyoxylate cycle intermediates citrate and succinate does not alter the efficiency of yeast CLS extension by LCA. WT cells and mutant cells carrying a single-gene-deletion mutation eliminating either the Cit2, Ctp1 or Dic1 protein were cultured in the nutrient-rich YP medium initially containing 0.2% glucose with 50 μM LCA or without it. **(A, B, C)** Survival curves of the chronologically aging WT and *cit2Δ* (A), WT and *ctp1Δ* (B) or WT and *dic1Δ* (C) strains are shown. Data are presented as means ± SEM (n = 3). Data for the WT strain cultured with or without LCA are replicated in graphs A, B, C of this Figure and in graphs A, B, C of Supplementary Figure 7. **(D, E, F)** *p* Values for different pairs of survival curves of the WT and *cit2Δ* (D), WT and *ctp1Δ* (E) or WT and *dic1Δ* (F) strains cultured with or without LCA. Survival curves shown in (A, B, or C, respectively) were compared. Two survival curves were considered statistically different if the *p* value was less than 0.05. The *p* values for comparing pairs of survival curves using the logrank test were calculated as described in Materials and Methods. The *p* values displayed on a yellow color background indicate that LCA extends the CLS of the WT, *cit2Δ* (D), *ctp1Δ* (E) and *dic1Δ* (F) strains. The *p* values displayed on a blue color background indicate that LCA extends the CLS of the *cit2Δ* (D), *ctp1Δ* (E) and *dic1Δ* (F) strains as efficiently as it extends the CLS of the WT strain. **(G, H)** Survival curves shown in (A, B, C) were used to calculate the fold of increase of the mean (G) and maximum (H) CLS by LCA for the WT, *cit2Δ*, *ctp1Δ* and *dic1Δ* strains. Data are presented as means ± SEM (n = 3; ns, not significant). Abbreviations: L, D, PD and ST, logarithmic, diauxic, post-diauxic and stationary growth phases (respectively).

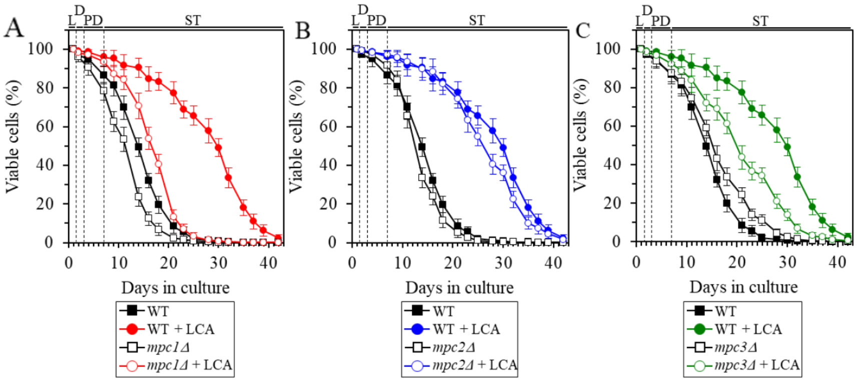

D	*p* Values for pairs of survival curves		
	WT + LCA	*mpc1Δ*	*mpc1Δ* + LCA
WT	**0.0012**	0.0634	0.0705
WT + LCA		**0.0005**	**0.0026**
mpc1Δ			**0.0418**

E	*p* Values for pairs of survival curves		
	WT + LCA	*mpc2Δ*	*mpc2Δ* + LCA
WT	**0.0012**	0.1176	**0.0023**
WT + LCA		**0.0008**	0.0629
mpc2Δ			**0.0017**

F	*p* Values for pairs of survival curves		
	WT + LCA	*mpc3Δ*	*mpc3Δ* + LCA
WT	**0.0012**	0.0732	**0.0119**
WT + LCA		**0.0018**	**0.0062**
mpc3Δ			**0.0431**

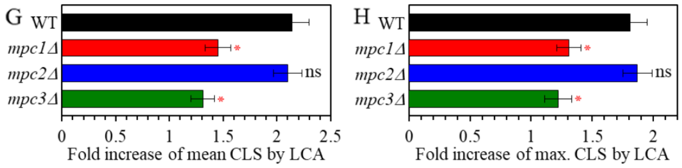

Supplementary Figure 7: Under CR conditions in the presence of LCA, lack of the Mpc1 or Mpc3 protein component of the Mpc1/Mpc3 mitochondrial pyruvate carrier decreases the efficiency of yeast CLS extension by LCA, whereas lack of the Mpc2 protein component of the Mpc1/Mpc2 mitochondrial pyruvate carrier does not affect such efficiency. WT cells and mutant cells carrying a single-gene-deletion mutation eliminating either the Mpc1, Mpc2 or Mpc3 protein were cultured in the nutrient-rich YP medium initially containing 0.2% glucose with 50 µM LCA or without it. **(A, B, C)** Survival curves of the chronologically aging WT and *mpc1Δ* (A), WT and *mpc2Δ* (B) or WT and *mpc3Δ* (C) strains are shown. Data are presented as means ± SEM (n = 3). Data for the WT strain cultured with or without LCA are replicated in graphs A, B, C of this Figure and in graphs A, B, C of Supplementary Figure 6. **(D, E, F)** *p* Values for different pairs of survival curves of the WT and *mpc1Δ* (D), WT and *mpc2Δ* (E) or WT and *mpc3Δ* (F) strains cultured with or without LCA. Survival curves shown in (A, B or C, respectively) were compared. Two survival curves were considered statistically different if the *p* value was less than 0.05. The *p* values for comparing pairs of survival curves using the logrank test were calculated as described in Materials and Methods. The *p* values displayed on a yellow color background indicate that LCA extends the CLS of the WT, *mpc1Δ* (D), *mpc2Δ* (E) and *mpc3Δ* (F) strains. The *p* values displayed on a blue color background in D and F indicate that LCA extends the CLS of the *mpc1Δ* (D) and *mpc3Δ* (F) strains to a lower extent than that of the WT strain. The *p* value displayed on a blue color background in E indicates that LCA extends the CLS of the *mpc2Δ* strain as efficiently as it extends the CLS of the WT strain. **(G, H)** Survival curves shown in (A, B, C) were used to calculate the fold of increase of the mean (G) and maximum (H) CLS by LCA for the WT, *mpc1Δ*, *mpc2Δ* and *mpc3Δ* strains. Data are presented as means ± SEM (n = 3; $^{*}p < 0.05$; ns, not significant). Abbreviations: L, D, PD and ST, logarithmic, diauxic, post-diauxic and stationary growth phases (respectively).

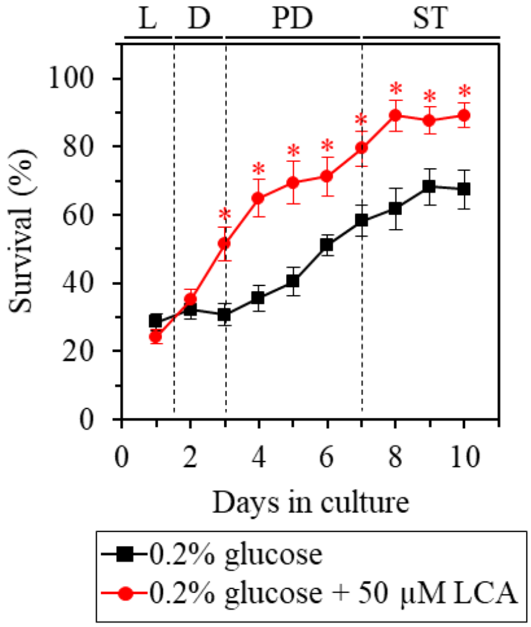

Supplementary Figure 8: Under CR conditions, LCA decreases cell susceptibility to mitochondria-controlled apoptotic RCD in an age-dependent manner. WT cells were cultured in the nutrient-rich YP medium initially containing 0.2% glucose with 50 μM LCA or without it. An assay for measuring clonogenic survival of cells recovered on different days of culturing and then treated for 2 h with 2.5 mM hydrogen peroxide to elicit a mitochondria-controlled mode of apoptotic RCD was performed as described in Materials and Methods. Data are presented as means ± SEM ($n = 3$; $^{*}p < 0.05$). Abbreviations: L, D, PD and ST, logarithmic, diauxic, post-diauxic and stationary growth phases (respectively).
